# Covalently integrated curcumin turns piperazine-based polyurethane-urea into a scaffold-as-drug for infected bone repair

**DOI:** 10.1016/j.mtbio.2026.103763

**Published:** 2026-10-20

**Authors:** Tao Yu, Jianxiang Zhu, Chenguang Cai, Jiawei Mei, Yong Xu, Liying Yang, Xiangyang Xu, Ming Ni, Qianming Li

**Affiliations:** a Department of Orthopaedics, Ruijin Hospital, Shanghai Jiao Tong University, School of Medicine, Shanghai, China; b Department of Orthopaedics, Ruijin-Hainan Hospital (Hainan Boao Research Hospital), Shanghai Jiao Tong University, School of Medicine, Qionghai, China; c Department of Orthopaedics, First Affiliated Hospital of Huzhou Normal University, Huzhou, China; d Graduate School of Bengbu Medical University, Bengbu, China; e Department of Orthopedics, Centre for Leading Medicine and Advanced Technologies of IHM, The First Affiliated Hospital of USTC, Division of Life Sciences and Medicine, University of Science and Technology of China, Hefei, China; f Department of Nursing, Ruijin Hospital, Shanghai Jiao Tong University, School of Medicine, Shanghai, China; g Department of Thoracic Surgery, Shanghai Pulmonary Hospital, School of Medicine, Tongji University, Shanghai, China

**Keywords:** Scaffold-as-drug, Curcumin, Bioactive polyurethane-urea scaffold, 3D printing, Tissue engineering

## Abstract

Infected bone defects are difficult to repair because bacterial persistence, excessive inflammation, oxidative stress, and impaired osteogenesis occur simultaneously. Conventional drug-loaded scaffolds often rely on physical loading, which can cause burst release and premature drug depletion. Here, we developed a scaffold-as-drug platform by covalently integrating curcumin (CUR) into a 3D-printable piperazine-based polyurethane-urea (P-PUU) backbone. CUR served as a reactive phenolic chain extender, while piperazine provided urea-forming structural segments. Spectroscopic and chromatographic analyses supported polymer formation and CUR incorporation, and molecular simulations indicated reduced chain mobility and stronger interchain association after CUR integration. The resulting CUR-P-PUU scaffolds retained interconnected porosity, improved apparent wettability, enhanced mechanical performance, and provided sustained degradation-associated CUR availability. Although physically blended CUR/P-PUU showed stronger short-term osteogenic responses *in vitro*, consistent with greater early CUR availability, CUR-P-PUU exhibited a more sustained CUR-availability profile during scaffold degradation. CUR-P-PUU supported BMSC viability, inhibited methicillin-resistant *Staphylococcus aureus* (MRSA), suppressed LPS-induced inflammatory cytokines, and promoted osteogenic differentiation. In a rat MRSA-infected calvarial defect model, CUR-P-PUU reduced bacterial burden, was associated with a less inflammatory and more pro-reparative local immune phenotype, and enhanced bone formation and mineralization. This study establishes covalent small-molecule integration as a materials-driven strategy for infection-resilient bone regeneration in a printable biodegradable scaffold.

## Highlights

• A scaffold-as-drug strategy was developed by covalently integrating curcumin into PUU.
• Curcumin acted as a bioactive chain extender to construct a therapeutic 3D-printed scaffold.
• Covalent CUR integration enabled sustained degradation-associated bioactive availability.
• CUR-P-PUU scaffold suppressed MRSA infection and modulated regenerative immunity.
• The scaffold promoted bone regeneration in an MRSA-infected defect model.

## Graphical abstract

**Figure:**
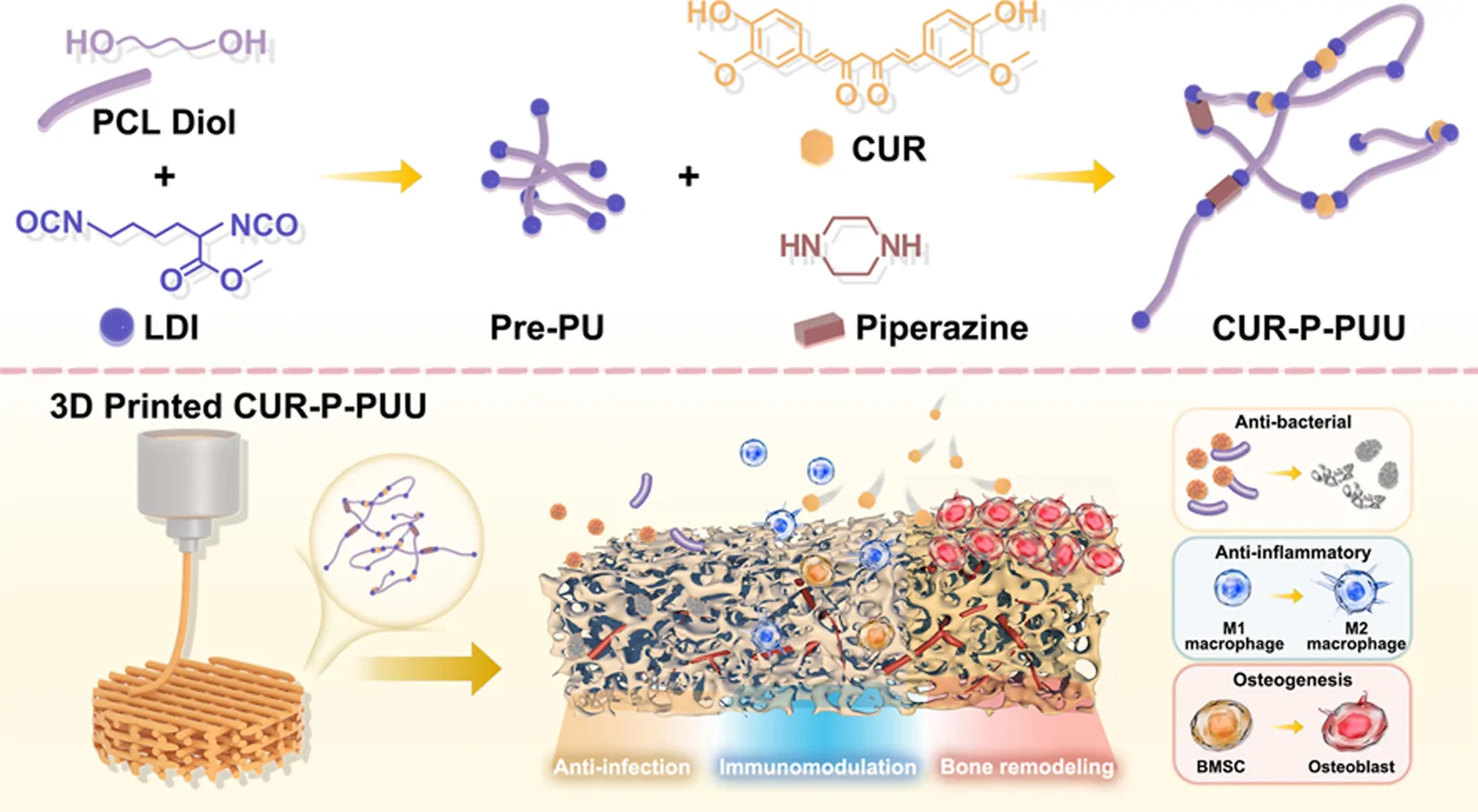

## 1 Introduction

Three-dimensional (3D) scaffolds for bone repair are no longer expected to serve only as passive space fillers [1]. An ideal scaffold should provide a temporary mechanical framework, guide cell infiltration and tissue ingrowth, degrade in a controlled manner, and deliver local biological cues during the repair process [2]. However, in many current designs, these functions are still assembled in a relatively loose way: a structural scaffold is first fabricated, and therapeutic agents are then physically blended, adsorbed, or coated onto the material [3]. Although this strategy is straightforward, the drug and the scaffold remain only weakly connected. As a result, local delivery is often governed mainly by diffusion, leading to early burst release, rapid loss of activity, and poor synchronization between material degradation and tissue regeneration [4]. This mismatch becomes particularly problematic when long-term and spatially stable bioactivity is required.

Infected bone defects represent a particularly demanding materials challenge because bacterial colonization, excessive inflammation, oxidative stress, and impaired osteogenesis coexist and mutually reinforce one another [5]. Accordingly, scaffolds designed for this condition must provide more than structural defect filling or short-term antibacterial delivery; ideally, they should maintain antibacterial activity, modulate the inflammatory microenvironment, alleviate oxidative stress, and support osteogenic differentiation throughout the repair process [6], [7]. To achieve such multifunctionality, a variety of scaffold-design strategies have been explored, including the incorporation of single or multiple therapeutic agents, bioactive nanoparticles, functional surface coatings, and layered or hybrid architectures [8]. These approaches can endow scaffolds with combined antibacterial, immunomodulatory, antioxidant, and osteogenic properties, but the structural matrix and therapeutic components often remain functionally distinct. In physically loaded systems, therapeutic performance may additionally be constrained by burst release and premature drug depletion. More complex multicomponent designs can further increase compositional and fabrication complexity, potentially complicating reproducibility, manufacturing, and translational development [9], [10]. These limitations highlight the need for more integrated material strategies in which structural support and therapeutic function are incorporated within the same scaffold-forming platform.

A more integrated materials strategy is to make the scaffold itself therapeutically active [11]. In this “scaffold-as-drug” concept, bioactive molecules are not simply entrapped within the scaffold; instead, they are covalently incorporated into the polymer backbone and become part of the material structure [12]. Such a design may shift drug availability from diffusion-dominated leakage toward more sustained degradation-associated availability. In principle, therapeutic availability may therefore become more closely associated with scaffold erosion, allowing local bioactivity to better follow the lifetime of the implant [13]. This approach also provides a direct way to couple molecular composition, mechanical behavior, degradation, and biological function within one material platform. Despite these advantages, it remains difficult to implement. The bioactive molecule must contain suitable reactive groups, tolerate polymerization conditions, retain biological activity after incorporation, and remain accessible in an active or functionally useful form as degradation proceeds. At the same time, its incorporation must not impair printability, porous architecture, mechanical integrity, or cytocompatibility [14].

Curcumin (CUR) is an attractive candidate for this type of molecular materials design [15]. As a natural polyphenol, CUR possesses antibacterial, antioxidant, anti-inflammatory, and osteogenic potential [16], [17]. More importantly from a polymer chemistry perspective, its phenolic hydroxyl groups can react with isocyanate-terminated polymers to form urethane linkages. This feature allows CUR to be used not merely as a loaded drug, but as a reactive bioactive chain extender. If properly incorporated, CUR can contribute both to the chemical architecture of the polymer and to the biological function of the final scaffold [18], [19]. However, this possibility has not been fully explored in printable biodegradable scaffolds for infected bone repair.

Polyurethane-urea (PUU) is particularly suitable for this purpose because its properties can be tuned through the balance between soft and hard segments [20]. Poly(ε-caprolactone) (PCL)-rich soft segments provide flexibility and degradability, whereas urethane- and urea-containing hard segments contribute to intermolecular association, mechanical strength, and structural stability [21]. In the present P-PUU system, piperazine functions as a urea-forming structural chain extender. Its reaction with isocyanate-terminated segments introduces piperazine-derived urea units into the polymer backbone, thereby contributing to the hard-segment architecture required for scaffold integrity and mechanical support. This structural role complements that of CUR, which is introduced as a bioactive phenolic chain extender [22].

Here, we report a CUR-integrated P-PUU scaffold designed as an integrated therapeutic scaffold in which bioactivity is built directly into the scaffold-forming polymer. Previous studies have demonstrated the feasibility of incorporating CUR into polyurethane through its reactive phenolic groups [23]. Building on this concept, we designed a distinct dual-chain-extension strategy in which CUR was covalently incorporated as a bioactive phenolic chain extender, while piperazine served as a urea-forming structural chain extender within the piperazine-based PUU (P-PUU) backbone. This dual-chain-extension strategy converts CUR from a physically loaded cargo into a molecular building block of the scaffold. We hypothesized that this covalent integration would reinforce intermolecular interactions, preserve printability and porous architecture, and provide sustained, degradation-associated CUR availability.

Although CUR has been incorporated into numerous bone-regenerative materials, most reported systems use CUR as a physically loaded therapeutic component, in which drug delivery and scaffold structure remain functionally separated. The innovation of the present strategy therefore lies not simply in introducing CUR into another bone scaffold, but in redefining its material role. By using CUR as a reactive chain-extending component of the P-PUU backbone, CUR becomes a structural and functional building block of the scaffold-forming polymer itself, rather than an independently loaded therapeutic cargo. This molecular integration is intended to couple CUR availability with the evolution of the biodegradable scaffold while simultaneously allowing CUR-derived structural units to influence polymer-chain interactions and material properties.

To test this hypothesis, we systematically investigated the chemical structure, molecular interactions, thermal and mechanical properties, degradation behavior, and CUR-availability profile of CUR-P-PUU scaffolds. We further evaluated whether this material-level design could translate into biological function, including cytocompatibility, radical-scavenging activity, anti-inflammatory regulation, antibacterial activity against methicillin-resistant *Staphylococcus aureus* (MRSA), and osteogenic differentiation of bone marrow mesenchymal stem cells (BMSCs). Finally, the therapeutic performance of CUR-P-PUU was assessed in a rat MRSA-infected calvarial defect model. By connecting polymer design with scaffold properties, degradation-associated CUR availability, and biological performance, this study evaluates covalent CUR integration as a materials-based strategy for infected bone repair (Fig. 1).

**Fig. 1:**
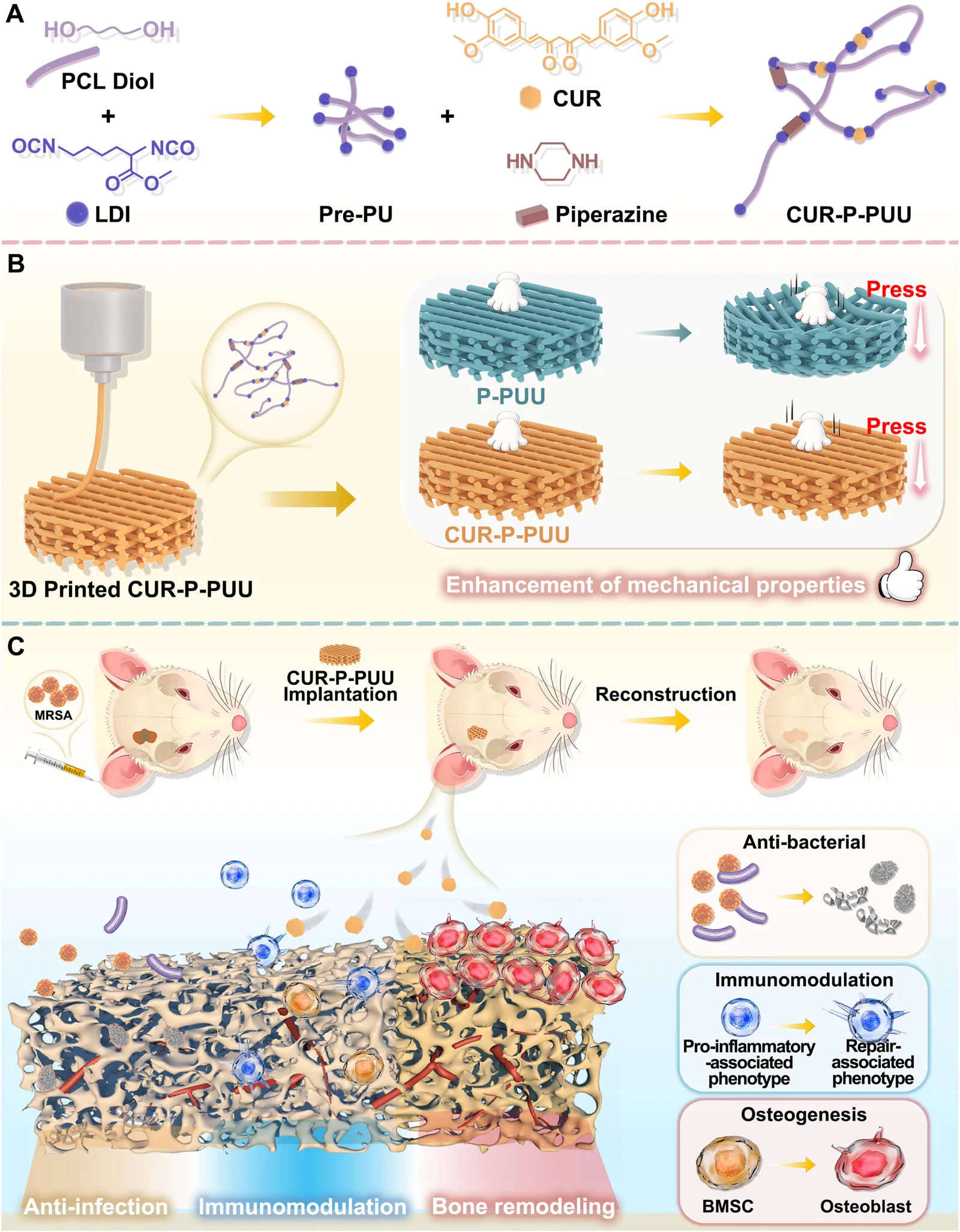
**Design rationale and therapeutic functions of 3D-printed CUR-P-PUU scaffolds for infected bone defect repair.** L-lysine diisocyanate (LDI) is first reacted with poly(ε-caprolactone) diol (PCL-diol) to form an NCO-terminated polyurethane prepolymer (Pre-PU). CUR is then introduced as a reactive phenolic chain extender, while piperazine serves as a urea-forming structural chain extender, yielding CUR-P-PUU in which CUR is covalently incorporated into the scaffold-forming polymer backbone. This design transforms CUR from a conventionally loaded therapeutic molecule into a structural and functional component of the polymer. The resulting polymer is subsequently processed into porous scaffolds by 3D printing and implanted into a rat MRSA-infected bone defect model. Based on the *in vitro* degradation and CUR-availability results, covalent integration is designed to provide sustained, degradation-associated CUR availability while preserving CUR-related biological activity. The resulting scaffold exhibits antibacterial, radical-scavenging, immunomodulatory, and osteogenic functions that collectively support infected bone repair.

## 2 Results and discussion

### 2.1 Molecular design and chemical characterization of CUR-P-PUU polymer

Previous work has established that the phenolic functionalities of CUR can be exploited for its chemical incorporation into polyurethane structures [23]. In the present study, we extended this concept to a piperazine-based PUU platform by using CUR and piperazine as complementary chain extenders. Poly(ε-caprolactone) diol (PCL-diol) was first reacted with L-lysine diisocyanate (LDI) to form an NCO-terminated polyurethane prepolymer. CUR was then introduced as a reactive phenolic chain extender, while piperazine was used as a urea-forming structural chain extender, resulting in a CUR-integrated piperazine-based PUU backbone (Supplementary Fig. 1). In this dual-chain-extension design, PCL provides the degradable soft segment, whereas piperazine acts as the primary urea-forming structural chain extender and contributes to the hard-segment architecture of the PUU backbone. These piperazine-derived urea segments support intermolecular association and scaffold structural integrity. CUR, in contrast, serves as a bioactive phenolic chain extender that becomes integrated into the same polymer backbone and further modifies chain-level interactions while providing therapeutic functionality.

Molecular simulations were first used to examine whether CUR incorporation could alter chain-level behavior. The single-chain and condensed-phase models provided a structural basis for comparing pristine P-PUU and CUR-P-PUU (Fig. 2A). The mean squared displacement (MSD) curves showed lower chain mobility in CUR-P-PUU than in P-PUU (Fig. 2B), suggesting that CUR incorporation restricted segmental motion. The radial distribution function (RDF) analysis further showed stronger interchain association in CUR-P-PUU (Fig. 2C). The simulations showed that CUR incorporation reduced polymer-chain mobility and strengthened interchain interactions. These changes are consistent with the improved thermal and mechanical performance of CUR-P-PUU.

**Fig. 2:**
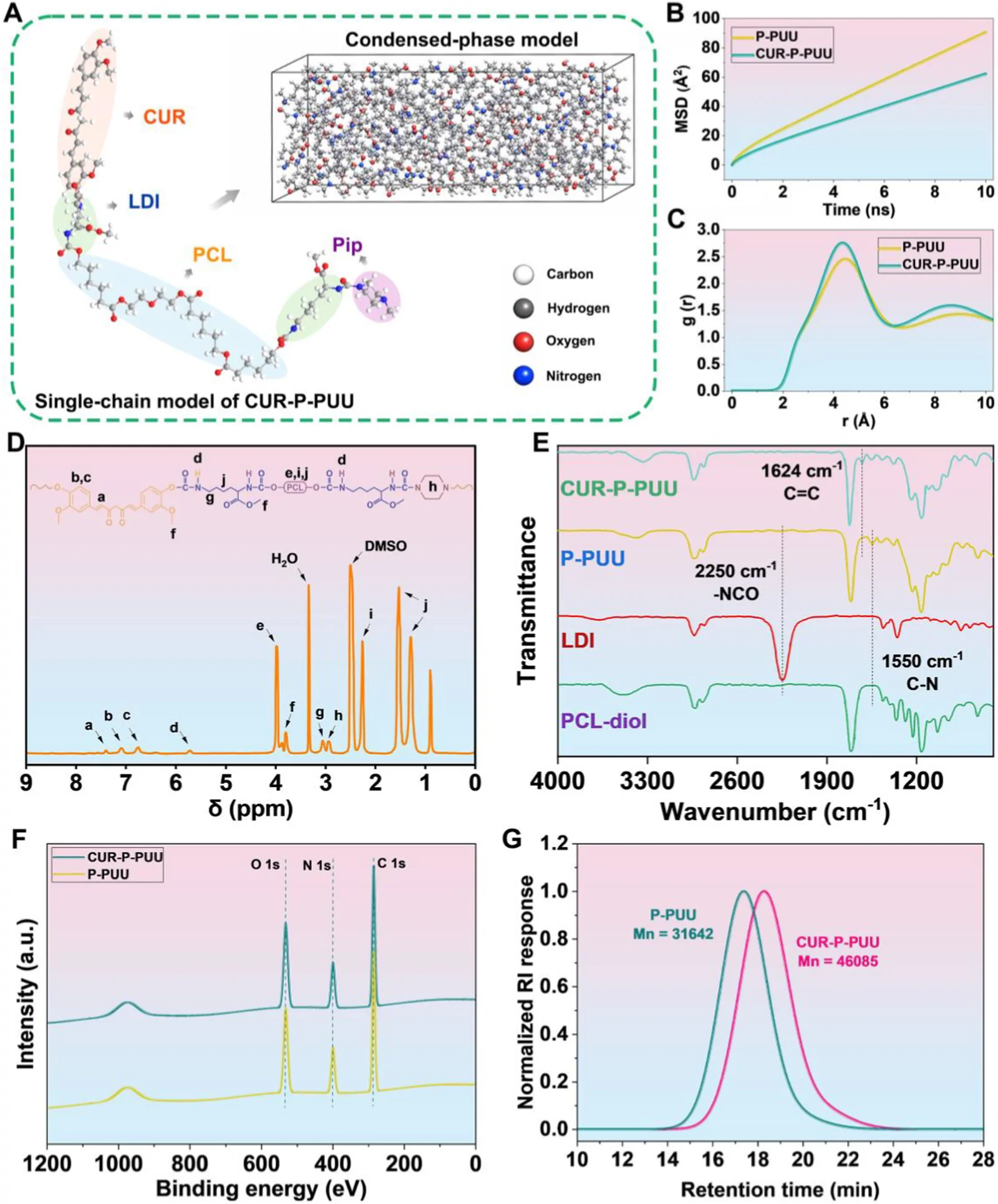
**Molecular design, simulation, and chemical characterization of CUR-P-PUU polymer.** (A) Representative molecular models of CUR-P-PUU, showing CUR incorporated as a chain-extending unit within the polymer structure, including single-chain and condensed-phase configurations. (B) Mean squared displacement (MSD) curves of polymer chains in P-PUU and CUR-P-PUU, used to compare polymer-chain mobility. (C) Total radial distribution functions (RDFs) between polymer chains in P-PUU and CUR-P-PUU, reflecting differences in interchain association. (D) ^1^H NMR spectrum of CUR-P-PUU. (E) FTIR spectra of LDI, PCL-diol, P-PUU, and CUR-P-PUU. (F) XPS spectra of P-PUU and CUR-P-PUU. (G) GPC chromatograms of P-PUU and CUR-P-PUU.

These molecular-level changes provide a plausible structural basis for the macroscopic behavior of CUR-P-PUU. Reduced segmental mobility and enhanced interchain association can increase resistance to chain slippage and improve stress transfer within the polymer network, which is consistent with the higher compressive modulus, improved cyclic compressive response, and increased tensile strength observed experimentally. At the same time, the segmented PUU architecture retains deformability through the PCL-rich soft domains, helping explain why mechanical reinforcement was achieved without a marked loss of elongation at break. The implications for degradation are more complex. Stronger interchain association may contribute to preservation of polymer and scaffold continuity, whereas the increased apparent wettability and water uptake after CUR incorporation may facilitate water penetration and enzymatic access. Thus, the final degradation behavior reflects a balance between enhanced structural cohesion and increased environmental accessibility rather than a simple monotonic effect of chain packing on degradation rate.

The successful synthesis and molecular incorporation of CUR in CUR-P-PUU were further verified by proton nuclear magnetic resonance spectroscopy (^1^H NMR), Fourier transform infrared spectroscopy (FTIR), X-ray photoelectron spectroscopy (XPS), and gel permeation chromatography (GPC). ^1^H NMR analysis provided further evidence for the formation of CUR-P-PUU (Fig. 2D and Supplementary Table S1). Signals observed in the 7.4, 7.1, and 6.7 ppm region were assigned to the aromatic and conjugated olefinic protons of CUR [24], while a weak resonance at approximately 5.7 ppm was attributed to the N-H proton of the carbamate moiety. The methoxy protons of CUR, together with the ester methoxy group of LDI, contributed to the signal at approximately 3.8 ppm [25]. Characteristic PCL and LDI-derived chain resonances were observed at approximately 3.9, 2.3, 1.5, and 1.3 ppm [26]. Signals at approximately 3.0 ppm were attributed to methylene groups adjacent to nitrogen atoms in the LDI-derived hard segments [27]. The signal at approximately 2.9 ppm was assigned to protons from the piperazine segment [28]. The simultaneous presence of characteristic signals from CUR, piperazine, PCL, and LDI-derived structures indicates that all designed molecular components were incorporated into the CUR-P-PUU polymer. Importantly, the presence of CUR-related proton signals in the polymer spectrum, together with the reduced free phenolic hydroxyl signature, supports the participation of CUR phenolic hydroxyl groups in urethane bond formation [18].

In the FTIR spectra (Fig. 2E), the characteristic isocyanate absorption band of LDI at approximately 2250 cm^−1^ disappeared in both P-PUU and CUR-P-PUU, indicating complete consumption of terminal NCO groups and sufficient progression of the chain-extension reaction. In P-PUU, a new amide-related absorption band appeared at approximately 1550 cm^−1^ after the reaction between piperazine and LDI, supporting the successful introduction of piperazine-derived urea segments into the polymer backbone. These urea-containing segments constitute part of the hard-domain architecture of P-PUU and provide a structural basis for intermolecular association and mechanical integrity. Compared with P-PUU, CUR-P-PUU showed an additional absorption band at approximately 1624 cm^−1^, which was assigned to the aromatic ring structure of CUR. This feature is consistent with the presence of CUR-derived aromatic moieties in CUR-P-PUU and, together with the other characterization results, supports CUR incorporation into the polymer structure.

XPS analysis further supported the surface elemental composition and bonding environments of P-PUU and CUR-P-PUU (Fig. 2F). The detected C, O, and N signals were consistent with the expected urethane/urea-containing polymer structure. High-resolution C 1s spectra were further deconvoluted into contributions assigned to C–C/C–H, C–O, C=O, and N–COO species (Supplementary Fig. 2). Notably, CUR-P-PUU additionally exhibited a π–π shake-up feature associated with aromatic/conjugated structures, consistent with the presence of CUR-derived aromatic moieties in the polymer. These XPS results support changes in the surface chemical environment following CUR incorporation; however, XPS alone does not constitute direct evidence of covalent bond formation. Therefore, the covalent integration of CUR was evaluated collectively based on the designed reaction chemistry, ^1^H NMR and FTIR characterization, and the low residual extractable CUR content after exhaustive purification.

GPC analysis showed a change in the apparent molecular-weight distribution after CUR incorporation (Fig. 2G). The calculated polystyrene-equivalent Mn values were 31,642 for P-PUU and 46,085 for CUR-P-PUU. Because hydrodynamic volume may vary with polymer composition, these values are interpreted as apparent molecular weights.

Taken together, these spectroscopic and chromatographic results support the successful incorporation of CUR into the P-PUU polymer structure. To further minimize the contribution of noncovalently retained CUR, the crude CUR-P-PUU reaction mixture was treated three times with methanol to remove unreacted or methanol-extractable CUR, followed by deionized-water-induced polymer precipitation and thorough washing of the collected polymer with deionized water. Residual extractable CUR in the purified polymer was subsequently quantified by HPLC after solvent extraction. The residual extractable CUR content was below 0.10 wt% of the purified CUR-P-PUU polymer, corresponding to less than 3% of the theoretical CUR content. The low level of solvent-extractable CUR indicates that only a small fraction of CUR remained in a freely extractable, noncovalently retained form after purification. Together with the ^1^H NMR and FTIR results and the consumption of residual NCO groups, this finding provides additional support for CUR incorporation into the P-PUU polymer structure.

### 2.2 Structural fidelity, mechanical behavior, degradation, and CUR availability of 3D-printed scaffolds

The synthesized polymers were processed into porous scaffolds by 3D printing. Gross photographs showed that pristine P-PUU scaffolds were pale, whereas CUR-P-PUU scaffolds showed a uniform yellow-orange color (Fig. 3A). This color change was consistent with homogeneous CUR distribution throughout the scaffold. Scanning electron microscopy (SEM) images showed that both P-PUU and CUR-P-PUU scaffolds maintained regular, interconnected porous architectures (Fig. 3B). Quantitative porosity analysis further showed no significant difference among P-PUU, physically blended CUR/P-PUU, and CUR-P-PUU scaffolds (Supplementary Fig. 3A). These results indicate that covalent CUR incorporation did not compromise print fidelity or macroporous architecture.

**Fig. 3:**
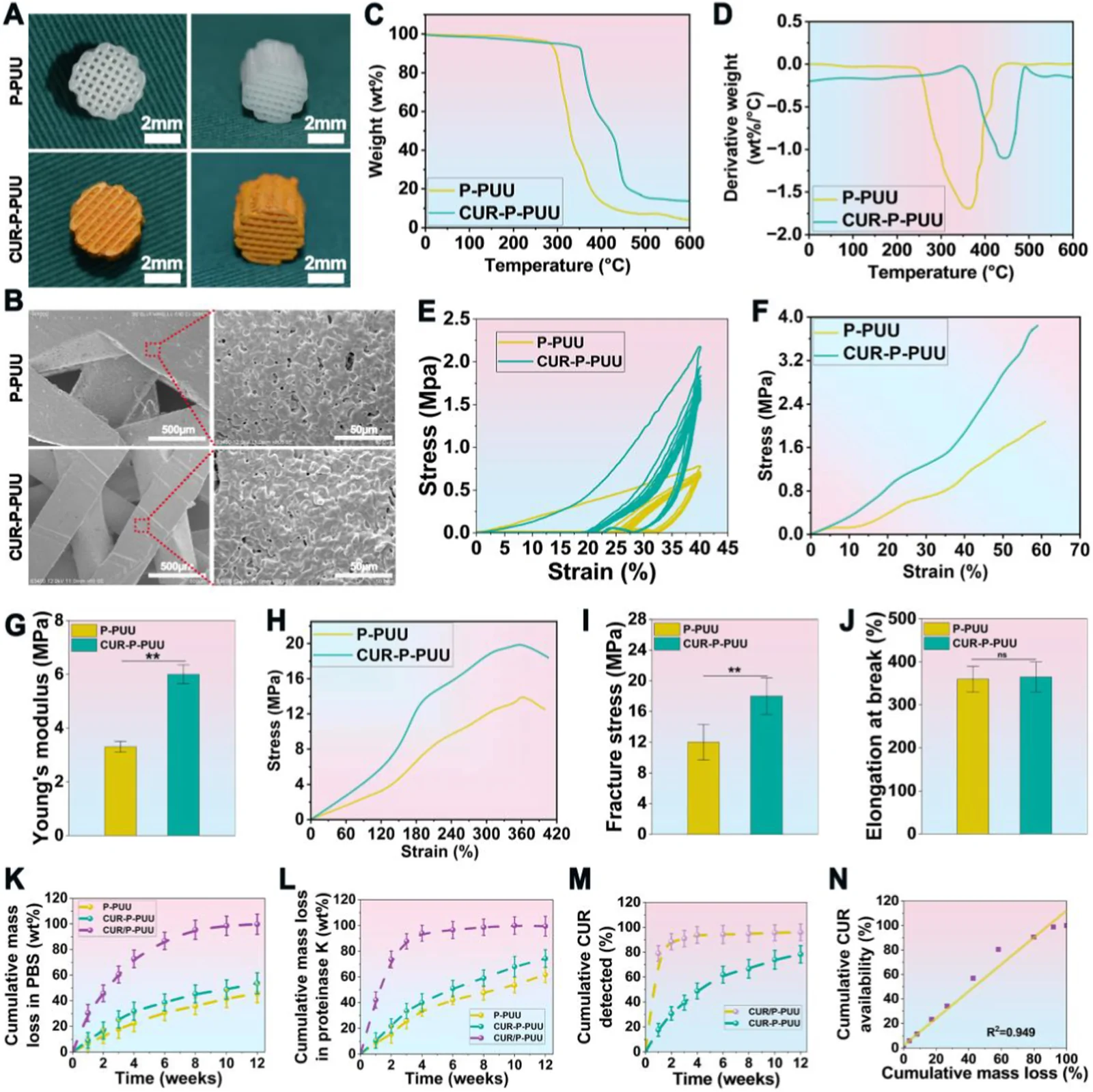
**Structural fidelity, mechanical performance, and degradation-associated CUR availability of 3D-printed CUR-P-PUU scaffolds.** (A) Representative photographs of 3D-printed P-PUU and CUR-P-PUU scaffolds. (B) SEM images of 3D-printed P-PUU and CUR-P-PUU scaffolds; regions outlined by red dashed boxes are shown at higher magnification. (C) TGA and (D) DTG curves of P-PUU and CUR-P-PUU scaffolds. (E) Cyclic compressive stress–strain curves of P-PUU and CUR-P-PUU scaffolds. (F) Compressive stress–strain curves and (G) compressive Young's modulus of P-PUU and CUR-P-PUU scaffolds. (H) Tensile stress–strain curves, (I) fracture stress, and (J) elongation at break of P-PUU and CUR-P-PUU scaffolds. Apparent cumulative mass loss in (K) PBS and (L) proteinase K of P-PUU, CUR-P-PUU, and physically blended CUR/P-PUU scaffolds. (M) Cumulative CUR detected in the degradation/release medium from CUR-P-PUU and physically blended CUR/P-PUU scaffolds. (N) Relationship between cumulative CUR detected in the medium and cumulative enzymatic mass loss of CUR-P-PUU under enzymatic degradation conditions. Each point represents the cumulative CUR detected in the medium plotted against the corresponding cumulative scaffold mass loss at the same degradation time point. The solid line represents the linear regression fit, with the coefficient of determination (R^2^) indicated in the panel. For quantitative mechanical analyses (G, I, J), n = 5 independent scaffold specimens per group; data are presented as mean ± SD and were analyzed using an unpaired two-tailed Student's *t*-test. For time-dependent degradation and CUR-detection analyses (K–M), *n* = 5 independent scaffold specimens per group; data are presented as mean ± SD and were analyzed using two-way ANOVA followed by Šídák's multiple-comparison test. ns, not significant; **P < 0.01. (For interpretation of the references to color in this figure legend, the reader is referred to the Web version of this article.)

Compared with pristine P-PUU, the CUR-containing scaffolds exhibited lower apparent water contact angles, with CUR-P-PUU showing the most pronounced decrease (Supplementary Fig. 3B). Although free CUR itself has poor aqueous solubility, water solubility and surface wettability are distinct properties. CUR contains multiple oxygen-containing functionalities capable of interfacial interactions with water, and their presentation within the polymer matrix can alter the local surface chemical environment without requiring CUR dissolution [29]. In addition, because the measurements were performed on porous 3D-printed scaffolds, the obtained values represent apparent contact angles influenced by both surface chemistry and scaffold microstructure. Consistent with this interpretation, water uptake was also increased in the CUR-containing scaffolds and was highest in CUR-P-PUU (Supplementary Fig. 3C). Therefore, these results are more appropriately interpreted as improved apparent wettability and overall water affinity following CUR incorporation rather than as evidence that CUR itself is water-soluble or intrinsically hydrophilic.

Thermal analysis showed that CUR-P-PUU had improved thermal stability compared with pristine P-PUU, as reflected by the thermogravimetric analysis (TGA) and derivative thermogravimetry (DTG) curves (Fig. 3C and D). This improvement is consistent with the more restricted chain motion and stronger interchain association observed in the molecular simulations [30]. These results suggest that CUR incorporation alters intermolecular organization within the polymer matrix. The mechanical behavior of CUR-P-PUU should be understood in the context of its segmented PUU architecture. Piperazine-derived urea segments form part of the hard-domain framework that provides baseline intermolecular cohesion and structural stability, whereas the PCL-rich soft segments preserve flexibility and deformability. Because piperazine is present in both P-PUU and CUR-P-PUU, the additional mechanical enhancement observed after CUR incorporation is more appropriately attributed to CUR-induced changes in chain mobility and interchain association superimposed on this piperazine-containing structural framework [31]. This result supports the material design because the scaffold must retain structural integrity during processing and early implantation while still being degradable over time.

Mechanical testing further showed that CUR-P-PUU achieved a more favorable balance between strength and elasticity. Under cyclic compression, CUR-P-PUU produced higher stress responses than P-PUU at the same strain level, indicating improved compressive resistance and elastic recovery (Fig. 3E). Monotonic compression tests showed a steeper stress–strain curve and a higher compressive modulus for CUR-P-PUU (Fig. 3F and G). Tensile testing also showed increased tensile strength while maintaining comparable elongation at break (Fig. 3H–J). These findings are consistent with the simulation-derived reduction in segmental mobility and stronger interchain association after CUR incorporation, which may reduce chain slippage and improve stress transfer between polymer segments. Meanwhile, the segmented PUU architecture retains deformability through the PCL-rich soft domains, which may explain the preservation of comparable elongation at break despite the increased strength [32]. This combination is important for bone-repair scaffolds because excessive softness may fail to provide local support, whereas excessive stiffness may impair mechanical compatibility with regenerating tissue [14].

The degradation behavior of the scaffolds was examined under hydrolytic and enzymatic conditions. In phosphate-buffered saline (PBS), pristine P-PUU showed the lowest cumulative mass loss, whereas physically blended CUR/P-PUU exhibited the highest gravimetric cumulative mass loss (Fig. 3K). Because CUR represented only approximately 3 wt% of the CUR/P-PUU formulation, direct leaching of CUR itself cannot account for the large mass loss observed at later time points. The gravimetric measurement reflects the recoverable dry mass of the porous scaffold and therefore may include not only polymer degradation and erosion but also loss of structural integrity and detached polymer fragments during incubation and sample recovery. Physical incorporation and subsequent loss of CUR may contribute to the formation of local defects and facilitate medium penetration; however, this should be regarded as a secondary factor promoting scaffold destabilization rather than as the direct source of the large mass loss. Accordingly, the high mass loss of CUR/P-PUU is interpreted as apparent scaffold mass loss associated with accelerated erosion/disintegration of the physically blended construct, rather than complete molecular degradation caused by CUR diffusion [33].

In contrast, CUR-P-PUU displayed an intermediate and more gradual mass-loss profile. The low level of extractable free CUR remaining after purification suggests that simple leaching of unbound CUR was unlikely to dominate its long-term behavior. Under proteinase K treatment, CUR-P-PUU showed greater cumulative mass loss than pristine P-PUU but lower apparent mass loss than physically blended CUR/P-PUU (Fig. 3L). This behavior may reflect a balance between increased water/enzymatic accessibility and preserved structural cohesion. Improved apparent wettability and water uptake may facilitate medium penetration, whereas covalent CUR integration and stronger interchain association may help maintain scaffold integrity and limit rapid disintegration. Together, these effects are consistent with the gradual mass-loss profile of CUR-P-PUU and its sustained, degradation-associated CUR availability.

The release profiles further highlighted the difference between physical blending and covalent integration. Physically blended CUR/P-PUU exhibited a relatively rapid initial release (Fig. 3M). Because CUR was physically dispersed within the P-PUU matrix without covalent immobilization, CUR located at or near the scaffold surface could be preferentially extracted into the release medium. Moreover, the presence of 0.2% (w/v) sodium dodecyl sulfate (SDS) enhanced CUR solubilization and helped maintain sink conditions, thereby sustaining the concentration gradient for outward CUR transport. Thus, the early release from CUR/P-PUU is better attributed to the combined effects of surface-associated CUR, lack of covalent constraint, and SDS-assisted solubilization/leaching rather than to inherently rapid diffusion of CUR in water. By contrast, CUR-P-PUU showed a more gradual increase in detectable CUR over 12 weeks, consistent with sustained, degradation-associated CUR availability from the covalently integrated polymer.

To further examine the relationship between scaffold degradation and CUR availability, cumulative CUR availability from CUR-P-PUU was plotted against the corresponding cumulative enzymatic mass loss at matched time points (Fig. 3N). Linear regression showed a strong positive association between the two variables (R^2^ = 0.949). Because both parameters evolve with incubation time, this relationship should be interpreted as descriptive evidence that CUR availability is associated with scaffold degradation and mass loss, rather than as proof of a specifically degradation-triggered or molecular-cleavage-controlled release mechanism. The low level of extractable free CUR after purification further suggests that simple leaching of noncovalently retained CUR is unlikely to dominate the long-term profile. However, the exact CUR-containing species generated during polymer degradation and the specific chemical cleavage events responsible for their formation were not determined in the present study. Accordingly, we refer to this behavior as a sustained, degradation-associated CUR-availability profile. Together with the low extractable free-CUR fraction, these findings support a more sustained CUR-availability profile for covalently integrated CUR-P-PUU during scaffold degradation.

It should be noted that the degradation and release experiments and the short-term *in vitro* biological assays were designed to answer different questions. The 12-week degradation/release study was used to evaluate the long-term degradation-associated CUR availability of CUR-P-PUU, whereas the 24-h antibacterial and anti-inflammatory assays mainly assessed the initial biological responsiveness of the scaffold. During the early incubation period, the total mass loss of CUR-P-PUU was limited; therefore, the short-term biological effects should not be interpreted as being driven by extensive bulk degradation. Instead, they may be attributed to a combination of surface-presented CUR-containing segments, limited early release of CUR or CUR-containing low-molecular-weight fragments, and contact-mediated interactions at the scaffold–cell or scaffold–bacteria interface. This interpretation is consistent with the stronger short-term osteogenic responses observed for physically blended CUR/P-PUU and with its greater early CUR availability in the release assay, whereas CUR-P-PUU provided a more sustained CUR-availability profile that may be more relevant to the longer *in vivo* repair process.

Taken together, these results provide the basis for distinguishing the three scaffold formulations used in the subsequent biological studies. P-PUU serves as the CUR-free scaffold control and represents the baseline contribution of the polymer matrix and scaffold architecture. In physically blended CUR/P-PUU, CUR remains noncovalently incorporated and is therefore more readily accessible at early time points through surface-associated release and leaching. In contrast, covalent incorporation in CUR-P-PUU restricts the immediate mobility of CUR but provides a more gradual, degradation-associated CUR-availability profile. Importantly, the short-term biological effects of CUR-P-PUU should not be attributed to extensive bulk degradation, which is limited during the early incubation period. Instead, early CUR-related activity may arise from surface-exposed CUR-containing segments, limited availability of CUR or CUR-containing low-molecular-weight species, and contact-mediated interactions at the scaffold–cell or scaffold–bacteria interface; as degradation proceeds, additional CUR-related species may become accessible and contribute to sustained biological activity. Thus, comparison of P-PUU, CUR/P-PUU, and CUR-P-PUU helps distinguish the contributions of the scaffold matrix, CUR bioactivity, and CUR incorporation mode, while also highlighting the trade-off between greater early CUR accessibility after physical loading and more sustained CUR availability after covalent integration. The exact chemical identity of the biologically active species generated during degradation was not determined in the present study; therefore, the CUR-P-PUU data are interpreted as evidence of sustained, degradation-associated CUR availability rather than a specific diffusion- or molecular-cleavage-controlled release mechanism.

### 2.3 Cytocompatibility of 3D-printed CUR-P-PUU scaffolds

Before evaluating therapeutic function, it was necessary to confirm that covalent CUR incorporation did not introduce cytotoxicity. BMSCs were cultured with P-PUU or CUR-P-PUU scaffolds for 1, 3, 5, and 7 days. Live/dead staining showed that viable cells predominated in all groups throughout the culture period, with only a small number of dead cells observed (Fig. 4A). F-actin staining further showed that BMSCs maintained normal cytoskeletal organization on the scaffolds (Fig. 4B). Over time, cells showed progressive spreading and more developed F-actin networks, indicating active attachment and growth. CUR-P-PUU did not impair this process. Instead, the improved apparent wettability and water uptake of CUR-P-PUU may have provided a more favorable interface for cell adhesion. These data indicate that CUR-P-PUU retained good cytocompatibility after chemical modification.

**Fig. 4:**
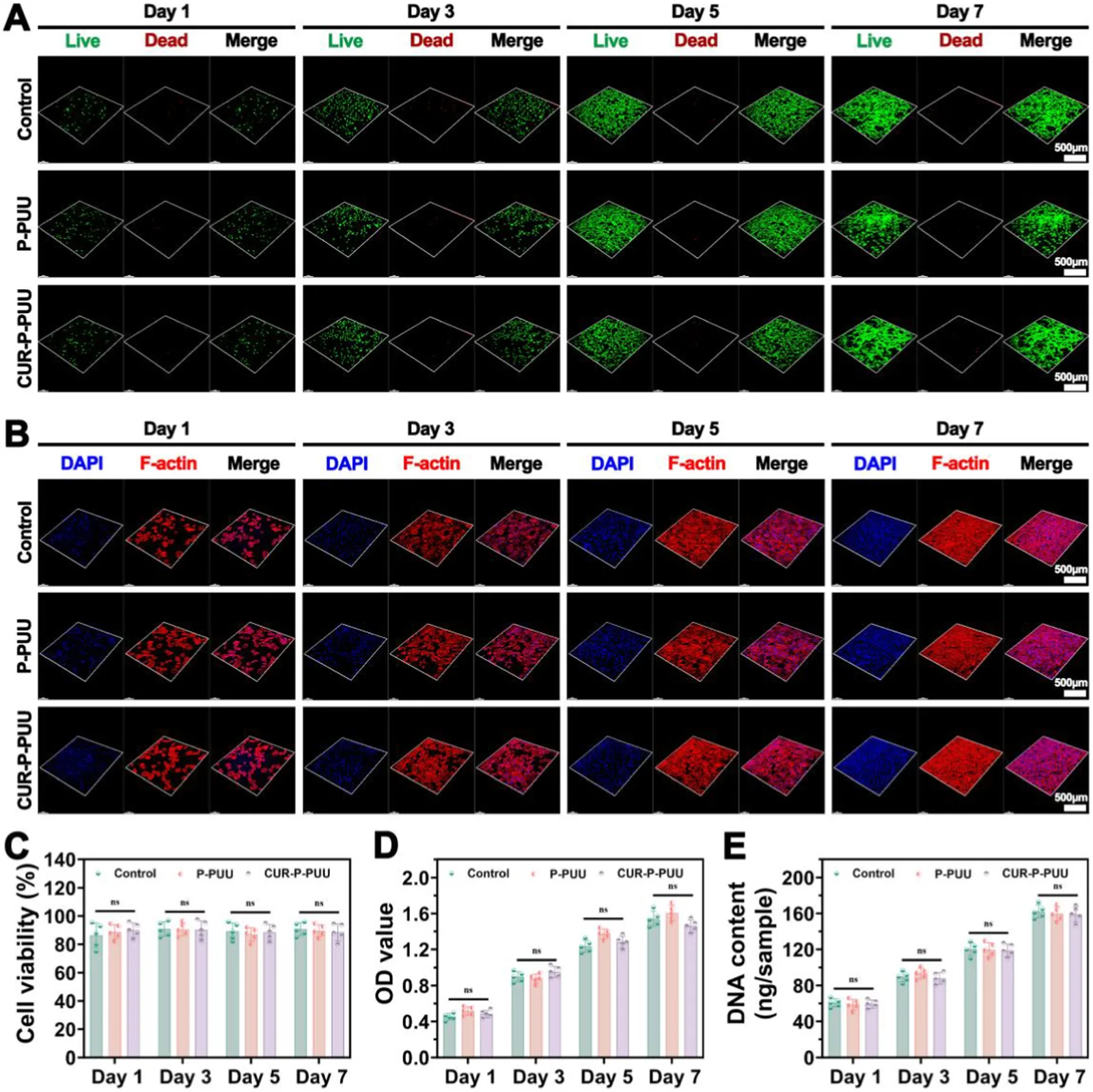
***In vitro* cytocompatibility of 3D-printed CUR-P-PUU scaffolds.** (A) Live/dead staining of BMSCs cultured with 3D-printed P-PUU or CUR-P-PUU scaffolds for 1, 3, 5, and 7 days; BMSCs cultured without scaffolds served as the control. (B) Fluorescence images of F-actin staining of BMSCs cultured with P-PUU or CUR-P-PUU scaffolds for 1, 3, 5, and 7 days; BMSCs cultured without scaffolds served as the control. (C) Quantification of cell viability in the control, P-PUU, and CUR-P-PUU groups based on live/dead staining. (D) Cell proliferation assessed by CCK-8 assay and expressed as optical density (OD) values at 1, 3, 5, and 7 days. (E) DNA content of BMSCs in the control, P-PUU, and CUR-P-PUU groups at 1, 3, 5, and 7 days. For quantitative analyses (C–E), *n* = 5 independent biological replicates per group; data are presented as mean ± SD. Statistical comparisons were performed using two-way ANOVA followed by Šídák's multiple-comparison test. ns, not significant.

Quantification of cell viability showed no significant difference among the control, P-PUU, and CUR-P-PUU groups (Fig. 4C). Cell Counting Kit-8 (CCK-8) assay analysis showed a time-dependent increase in cell metabolic activity from day 1 to day 7 (Fig. 4D), and DNA quantification showed a similar increase in cell number (Fig. 4E). No significant inhibitory effect was observed in the CUR-P-PUU group. These results show that the covalent integration of CUR did not compromise the basic cell-supportive function of the P-PUU scaffold, supporting its use in subsequent functional assays.

### 2.4 *In vitro* anti-inflammatory, radical-scavenging, and antibacterial activities

Infected bone defects are characterized by bacterial persistence and excessive inflammation. Therefore, the scaffold was next evaluated for its ability to modulate inflammatory responses and inhibit MRSA. RAW264.7 macrophages were stimulated with lipopolysaccharide (LPS) to establish an inflammatory model. Immunofluorescence staining showed that LPS markedly increased the expression of interleukin-6 (IL-6), interleukin-1 beta (IL-1β), and tumor necrosis factor-alpha (TNF-α) compared with the unstimulated control group (Fig. 5A). P-PUU alone did not substantially reduce these inflammatory signals under LPS stimulation. In contrast, CUR-containing groups, especially CUR-P-PUU + LPS and CUR/P-PUU + LPS, showed reduced cytokine fluorescence intensities (Fig. 5A and B). These results indicate that CUR retained anti-inflammatory activity after incorporation into the scaffold system.

**Fig. 5:**
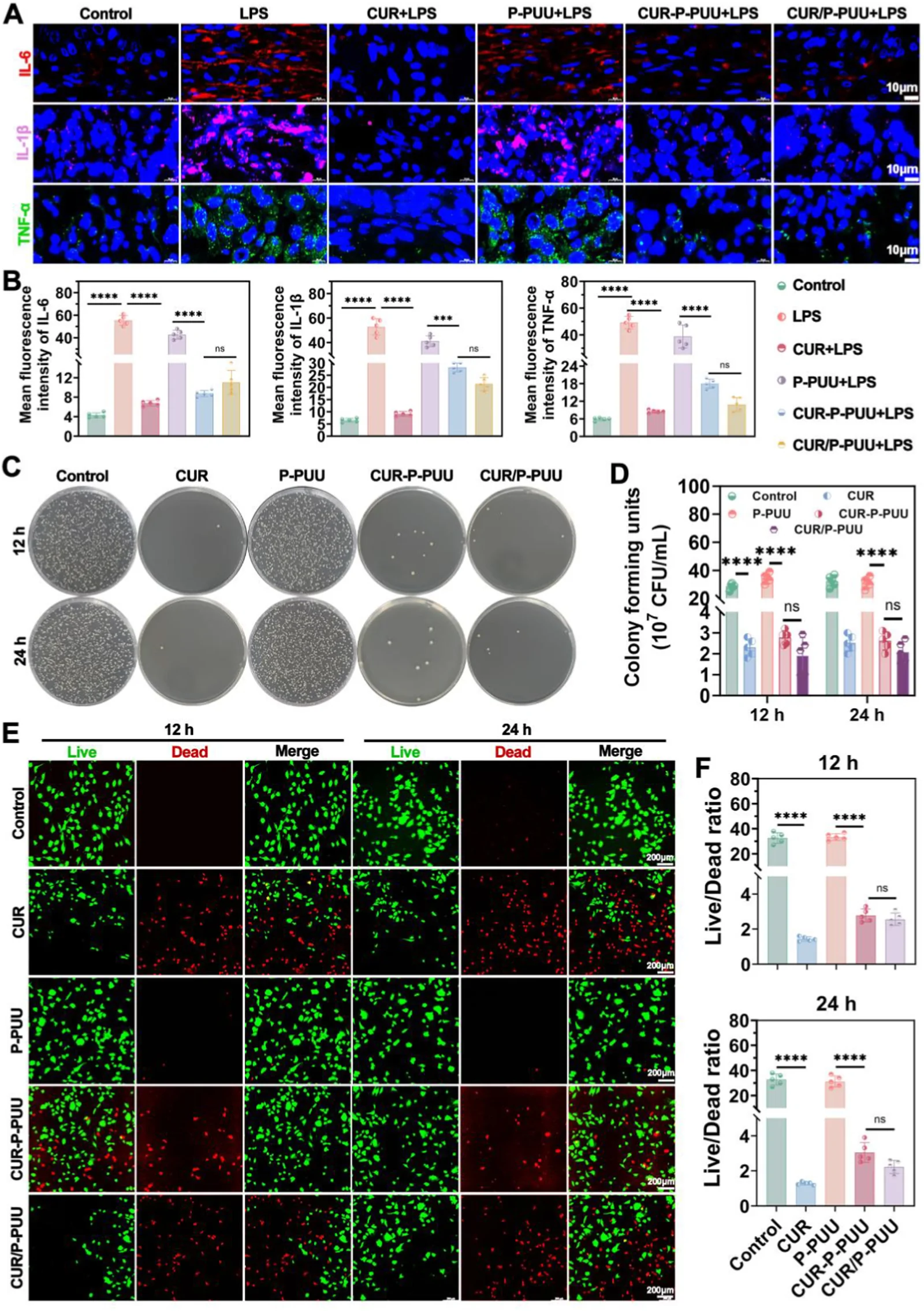
***In vitro* anti-inflammatory and antibacterial activities of 3D-printed CUR-P-PUU scaffolds.** (A) Immunofluorescence staining of IL-6, IL-1β, and TNF-α in LPS-stimulated RAW264.7 macrophages from the Control, LPS, CUR + LPS, P-PUU + LPS, CUR-P-PUU + LPS, and CUR/P-PUU + LPS groups. Cell nuclei were counterstained with DAPI (blue), while IL-6, IL-1β, and TNF-α were visualized in red, magenta, and green, respectively. (B) Semi-quantitative analysis of the mean fluorescence intensities of IL-6, IL-1β, and TNF-α in the groups shown in (A). (C) Representative agar plate images of MRSA colonies after 12 and 24 h of co-culture with the indicated groups: Control, free CUR, P-PUU, CUR-P-PUU, and CUR/P-PUU. (D) Planktonic bacterial colony-forming unit (CFU) counts after 12 and 24 h of co-culture. (E) Live/dead staining of planktonic MRSA collected after 12 and 24 h of co-culture with the indicated groups. Live bacteria were stained with SYTO 9 and are shown in green, whereas dead or membrane-compromised bacteria were stained with propidium iodide and are shown in red. (F) Quantification of the live/dead ratio of planktonic MRSA in the groups shown in (E). *n* = 5 independent biological replicates per group; data are presented as mean ± SD. For panel B, each cytokine was analyzed separately using one-way ANOVA followed by Tukey's multiple-comparison test. For panels D and F, statistical comparisons were performed using two-way ANOVA followed by Šídák's multiple-comparison test. ns, not significant; ***P < 0.001; ****P < 0.0001. (For interpretation of the references to color in this figure legend, the reader is referred to the Web version of this article.)

The macrophage cytoskeleton also changed after LPS stimulation. In the control group without LPS, RAW264.7 macrophages showed relatively regular morphology. After LPS stimulation, cells displayed cytoskeletal remodeling, including enhanced spreading and more obvious F-actin-rich protrusions (Supplementary Fig. 4). These changes are consistent with inflammatory activation. Compared with the LPS and P-PUU + LPS groups, CUR-containing groups showed relatively less activated morphology, consistent with reduced inflammatory activation in the presence of CUR-containing scaffolds. Although cell morphology alone cannot define macrophage phenotype, it provides supporting evidence that CUR-containing scaffolds can reduce inflammatory activation at the cellular level.

Because oxidative stress is closely linked to inflammation and impaired bone repair, the radical-scavenging activity of scaffold degradation media was assessed by 2,2′-azino-bis(3-ethylbenzothiazoline-6-sulfonic acid) (ABTS) radical-scavenging assay. Pristine P-PUU showed minimal radical-scavenging activity, whereas CUR/P-PUU and CUR-P-PUU both showed measurable radical-scavenging activity (Supplementary Fig. 5A). The time-dependent results were particularly informative. Physically blended CUR/P-PUU showed strong early radical-scavenging activity but decreased rapidly over time, consistent with early CUR release and depletion. In contrast, CUR-P-PUU maintained a more stable ABTS radical-scavenging activity over the 12-week period (Supplementary Fig. 5B). This result matches the release data and is consistent with the sustained CUR-availability profile observed during scaffold degradation. Instead of providing a short burst of radical-scavenging capacity, CUR-P-PUU provides sustained radical-scavenging capacity during scaffold degradation.

The antibacterial performance of the scaffolds was evaluated against MRSA. Agar plate images showed abundant bacterial colonies in the control and P-PUU groups after 12 and 24 h, whereas free CUR, CUR/P-PUU, and CUR-P-PUU markedly reduced colony formation (Fig. 5C). Planktonic colony-forming unit (CFU) counting showed reduced viable MRSA counts in the CUR-containing groups (Fig. 5D). Live/dead staining of planktonic MRSA showed a similar trend, with a greater proportion of dead or membrane-compromised bacteria in the CUR-containing groups (Fig. 5E and F). These results indicate that the antibacterial activity was predominantly associated with the presence of CUR, because P-PUU alone showed limited antibacterial effects whereas all CUR-containing groups reduced bacterial burden. These assays evaluated planktonic MRSA viability and did not directly assess scaffold-associated bacterial adhesion or mature biofilm formation; therefore, anti-biofilm efficacy remains to be established.

The difference between CUR/P-PUU and CUR-P-PUU should be interpreted in the context of their distinct CUR-availability profiles. The short-term antibacterial activity of physically blended CUR/P-PUU may be associated with greater early CUR availability, as noncovalently incorporated CUR is more readily accessible during the initial incubation period. Although this interpretation is consistent with the observed release profile, the actual CUR concentration in the antibacterial assay medium was not directly quantified, and the CUR dose was not systematically optimized. Therefore, the enhanced short-term response of CUR/P-PUU should not be considered evidence of an optimal antibacterial CUR dose. By contrast, CUR-P-PUU exhibited more gradual, degradation-associated CUR availability. Thus, its principal advantage may not be fully captured by short-term *in vitro* antibacterial assays, but rather lies in sustaining CUR-related antibacterial, radical-scavenging, and anti-inflammatory functions over a prolonged period during scaffold degradation.

### 2.5 *In vitro* pro-osteogenic effects of CUR-P-PUU scaffolds

The pro-osteogenic potential of the scaffolds was evaluated using BMSCs under osteogenic induction. Alkaline phosphatase (ALP) staining was used to assess early osteogenic differentiation [34]. After 7 days, both CUR-containing groups showed stronger ALP staining than the control and P-PUU groups, indicating that CUR contributed to early osteogenic commitment (Fig. 6A). Quantitative analysis further supported increased ALP activity in the CUR/P-PUU and CUR-P-PUU groups (Fig. 6B). Notably, the physically blended CUR/P-PUU group showed higher ALP activity than the CUR-P-PUU group. Matrix mineralization was then assessed by Alizarin Red S (ARS) staining after 21 days. Compared with the control and P-PUU groups, CUR-containing scaffolds exhibited more extensive ARS staining and mineralized matrix formation (Fig. 6C). Cetylpyridinium chloride (CPC)-based extraction and spectrophotometric quantification further confirmed significantly increased matrix-bound ARS content in the CUR-containing groups (Fig. 6D). Similar to the ALP results, the physically blended CUR/P-PUU group showed the most pronounced mineralization *in vitro*, whereas CUR-P-PUU also significantly enhanced mineralized matrix formation compared with the control and P-PUU groups. These results indicate that CUR is an effective pro-osteogenic cue in this material system. They also show that, under short-term *in vitro* osteogenic culture, greater early CUR availability may be associated with a stronger apparent osteogenic response than that associated with the more gradual CUR availability of covalently integrated CUR-P-PUU.

**Fig. 6:**
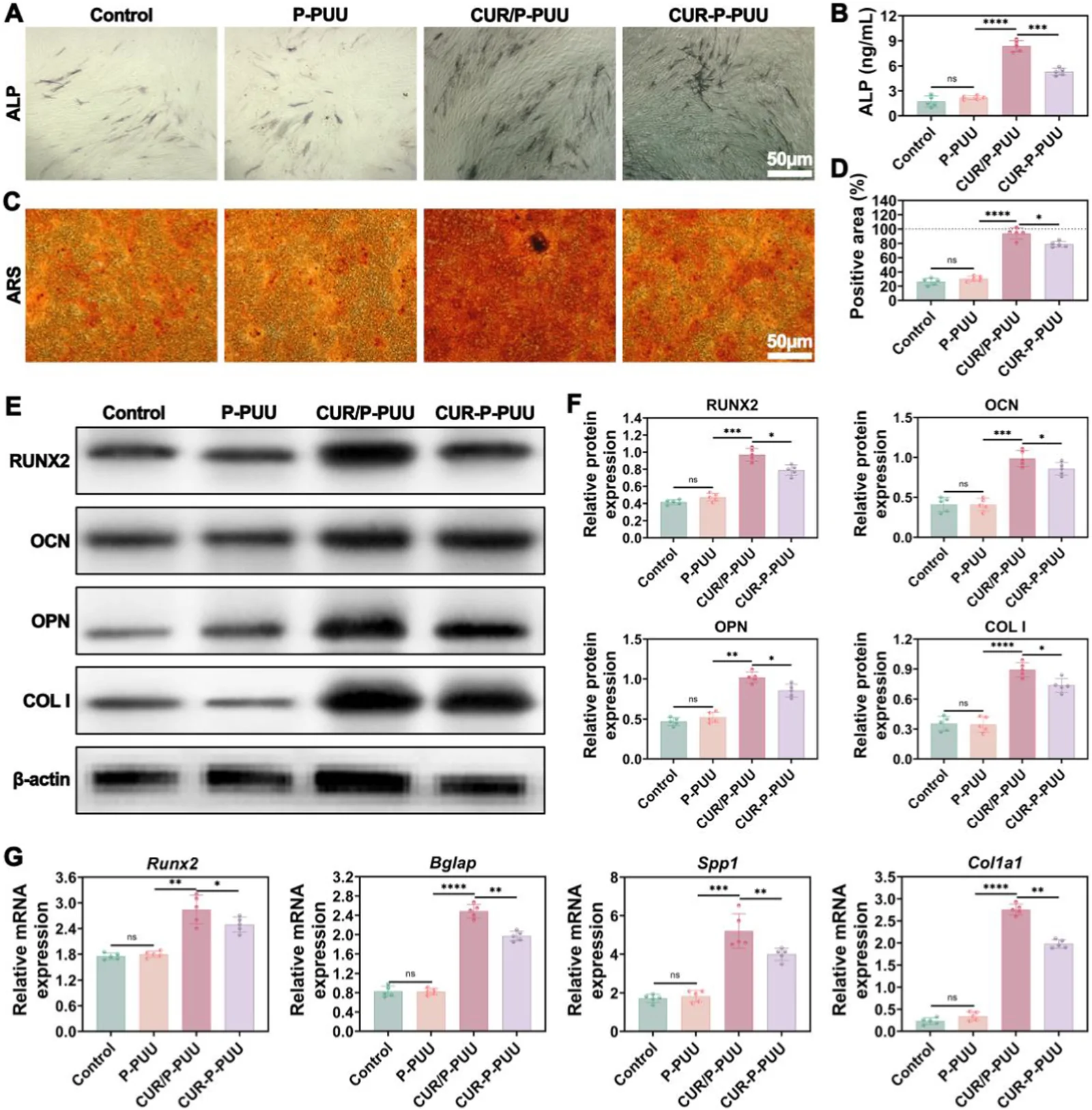
***In vitro* pro-osteogenic effects of 3D-printed CUR-P-PUU scaffolds.** (A) ALP staining of BMSCs in the control, P-PUU, CUR/P-PUU, and CUR-P-PUU groups after 7 days of osteogenic induction. (B) Quantification of ALP activity in the groups shown in (A). (C) ARS staining of mineralized matrix formed by BMSCs in the control, P-PUU, CUR/P-PUU, and CUR-P-PUU groups after 21 days of osteogenic induction. (D) Quantification of matrix mineralization by CPC extraction of matrix-bound ARS followed by spectrophotometric measurement at 562 nm. (E) Representative western blots and (F) densitometric quantification of osteogenesis-related proteins, including RUNX2, OCN, OPN, and COL I, in the indicated groups. (G) RT–qPCR analysis of osteogenic gene expression, including *Runx2*, *Bglap*, *Spp1*, and *Col1a1*, in the indicated groups. For quantitative analyses (B, D, F, G), *n* = 5 independent biological replicates per group; data are presented as mean ± SD. Statistical comparisons among groups were performed using one-way ANOVA followed by Tukey's multiple-comparison test. Each protein or gene was analyzed separately. ns, not significant; *P < 0.05; **P < 0.01; ***P < 0.001; ****P < 0.0001.

Western blot analysis further supported these findings. The expression levels of osteogenesis-related proteins, including runt-related transcription factor 2 (RUNX2), osteocalcin (OCN), osteopontin (OPN), and collagen type I (COL I), were increased in CUR-containing groups compared with the control and P-PUU groups (Fig. 6E and F). The physically blended CUR/P-PUU group generally showed stronger upregulation than CUR-P-PUU, in agreement with the ALP and ARS results. Reverse transcription quantitative polymerase chain reaction (RT–qPCR) analysis showed similar trends at the gene level, with increased expression of *Runx2*, *Bglap*, *Spp1*, and *Col1a1* in CUR-containing groups (Fig. 6G). RUNX2 is a key transcription factor for osteoblast differentiation, while COL I and OPN are closely associated with matrix formation and maturation. OCN is commonly linked to late-stage osteogenic differentiation and mineralization [35], [36]. Therefore, the coordinated changes in these osteogenesis-associated markers support enhanced osteogenic differentiation and matrix maturation in CUR-containing groups.

The stronger short-term osteogenic responses observed for physically blended CUR/P-PUU should be interpreted cautiously in relation to its release behavior. The release study showed greater early CUR availability from CUR/P-PUU than from covalently integrated CUR-P-PUU, which may contribute to the enhanced ALP activity, mineralization, and osteogenic marker expression observed during the relatively short *in vitro* culture period. However, the actual CUR concentration to which BMSCs were exposed was not directly quantified under the osteogenic culture conditions, and a systematic CUR dose–response optimization was not performed. Therefore, these results demonstrate an association between the physical incorporation mode, greater early CUR availability, and stronger short-term osteogenic responses, but they do not establish that the effect resulted from an optimized CUR dose or define a causal concentration–response relationship.

Thus, the apparently different performance of CUR/P-PUU and CUR-P-PUU *in vitro* and *in vivo* reflects a balance between immediate biological stimulation and long-term therapeutic sustainability. Physically blended CUR/P-PUU provides greater early CUR availability and was associated with a stronger short-term osteogenic response under *in vitro* culture conditions. In contrast, covalent integration limits rapid CUR loss and provides a more gradual, degradation-associated availability profile. Although this may reduce the magnitude of the initial osteogenic stimulus, it may be more advantageous during the prolonged course of infected bone repair, where sustained antibacterial, immunomodulatory, radical-scavenging, and osteogenic support is required. Consistent with this interpretation, CUR-P-PUU showed lower bacterial burden at 1 week, a less inflammatory and more pro-reparative local immune phenotype at 6 weeks, and enhanced bone regeneration *in vivo*. Nevertheless, because CUR concentrations under the osteogenic culture conditions and *in vivo* CUR availability over time were not directly quantified, this relationship should be regarded as an association between temporal CUR availability and biological performance rather than definitive evidence of a causal dose–response mechanism.

### 2.6 *In vivo* bone regeneration in a rat MRSA-infected calvarial defect model

The *in vivo* regenerative performance of CUR-P-PUU was evaluated in a rat MRSA-infected bone defect model, as illustrated in Supplementary Fig. 6. Micro-computed tomography (Micro-CT) 3D reconstructions showed limited bone regeneration in the control group at both 6 and 12 weeks after implantation (Fig. 7A and B). P-PUU showed some improvement compared with the empty defect, consistent with a beneficial contribution of the printed scaffold framework to bone repair. CUR/P-PUU further improved bone formation, suggesting that CUR bioactivity contributed to regeneration. However, CUR-P-PUU showed the most obvious defect filling and the most continuous bone bridging among all groups.

**Fig. 7:**
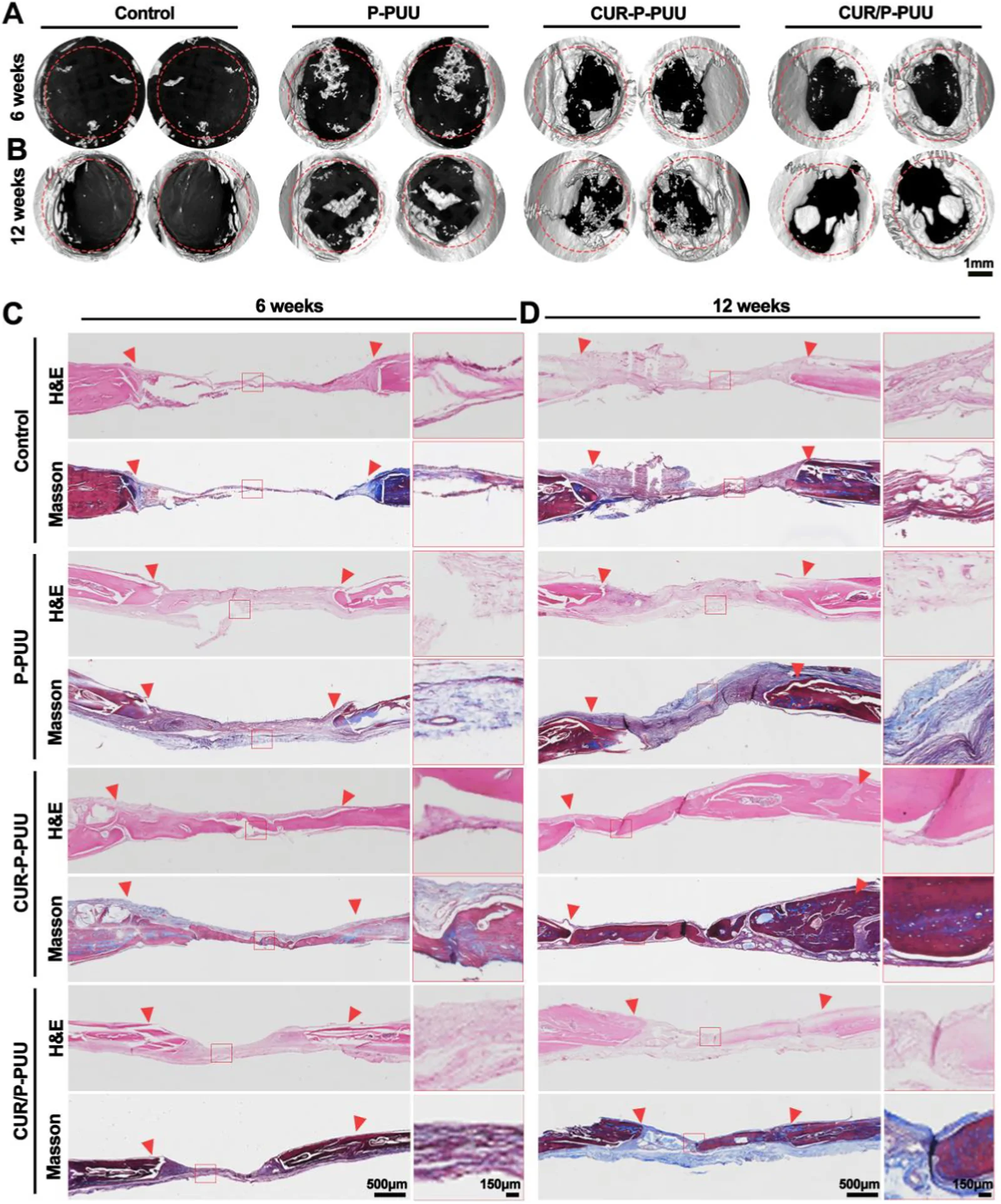
***In vivo* bone regeneration with 3D-printed CUR-P-PUU scaffolds in a rat MRSA-infected calvarial defect model.** (A, B) Representative micro-CT 3D reconstructions of defect sites in the control, P-PUU, CUR-P-PUU, and CUR/P-PUU groups at (A) 6 weeks and (B) 12 weeks after implantation. Red dotted circles indicate the original defect regions. (C, D) Representative histological images of defect-site tissue sections stained with H&E and Masson's trichrome in the control, P-PUU, CUR-P-PUU, and CUR/P-PUU groups at (C) 6 weeks and (D) 12 weeks after implantation. Red arrows indicate the regenerated regions. Images on the right show magnified views of the boxed regions in the corresponding histological sections. (For interpretation of the references to color in this figure legend, the reader is referred to the Web version of this article.)

Quantitative micro-CT analysis supported these observations. Bone volume/tissue volume (BV/TV) and bone mineral density (BMD) were increased in the CUR-P-PUU group compared with the control, P-PUU, and CUR/P-PUU groups at 6 and 12 weeks (Supplementary Fig. 7A–B). The increase in BV/TV indicates a greater amount of newly formed bone, while the increase in BMD suggests improved mineralization. Together, the higher BV/TV and BMD indicate greater bone volume and higher mineral density in the CUR-P-PUU group.

Histological analysis provided further evidence of bone repair. Hematoxylin and eosin (H&E) staining showed limited tissue ingrowth in the control group, whereas scaffold-treated groups showed increased cellular infiltration and new tissue formation within the defect region (Fig. 7C and D). CUR-P-PUU showed more abundant regenerated tissue and better continuity with the surrounding host bone. Masson's trichrome staining showed stronger collagen deposition in the CUR-P-PUU group, indicating active extracellular matrix formation and remodeling. The magnified views of the boxed regions further highlighted newly regenerated areas, and red arrows indicated the regenerated regions. These histological results are consistent with the micro-CT findings.

The *in vivo* results support the central material hypothesis of this study. In an infected defect, bone repair depends not only on osteogenic stimulation but also on infection control and inflammatory regulation. P-PUU can provide a printable and biodegradable structural framework, but it has limited intrinsic ability to modify the infected microenvironment. Physically blended CUR/P-PUU provides CUR bioactivity, but its early burst release may limit long-term effectiveness. CUR-P-PUU combines the structural advantages of P-PUU with sustained, degradation-associated CUR availability. These findings are consistent with the superior bone regeneration observed for CUR-P-PUU compared with P-PUU and physically blended CUR/P-PUU.

### 2.7 *In vivo* antibacterial and immunomodulatory effects

To determine whether the improved bone regeneration was associated with infection control, bacteria recovered from peri-implant tissues were cultured 1 week after implantation. Agar plates showed obvious MRSA colony growth in the control and P-PUU groups, whereas both CUR-containing scaffolds reduced bacterial recovery (Fig. 8A). CFU counting further supported that CUR/P-PUU and CUR-P-PUU significantly lowered the local bacterial burden compared with the control and P-PUU groups (Fig. 8B). Notably, CUR-P-PUU exhibited a significantly stronger antibacterial effect than physically blended CUR/P-PUU, as reflected by the lowest CFU counts among all groups. These results demonstrate that CUR-P-PUU significantly reduced the local MRSA burden after implantation and showed stronger *in vivo* antibacterial activity than physically blended CUR/P-PUU. However, the residual CFU detected in peri-implant tissues indicates suppression rather than complete eradication of infection.

**Fig. 8:**
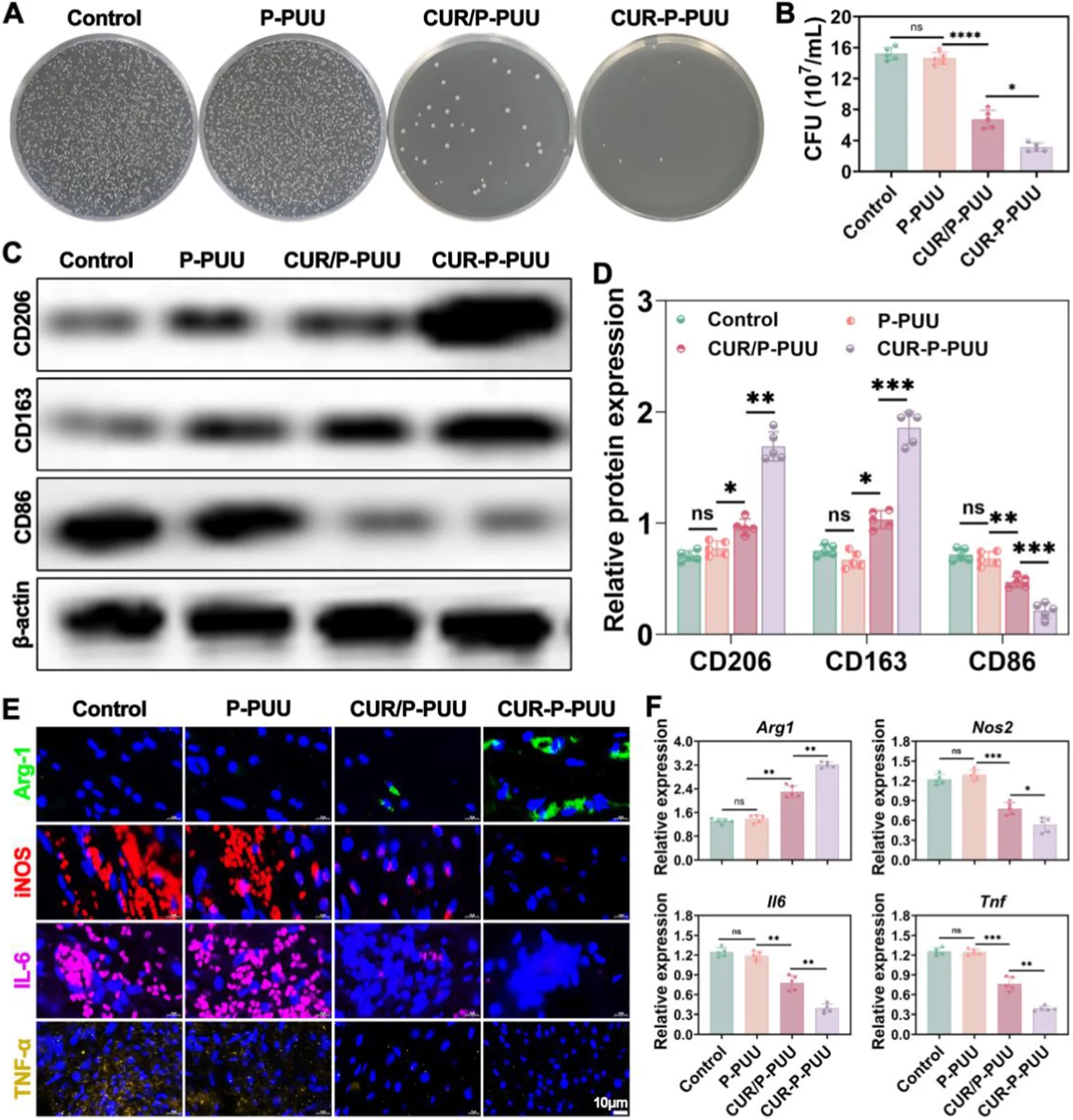
***In vivo* antibacterial and immunomodulatory effects of 3D-printed CUR-P-PUU scaffolds.** (A) Representative agar plates showing bacterial colonies recovered from peri-defect tissues in the control group and peri-implant tissues in the scaffold-treated groups 1 week after implantation. (B) Quantification of bacterial burden by CFU counting in the groups shown in (A). (C) Representative western blots of macrophage phenotype-associated markers in peri-implant tissues collected 6 weeks after implantation, including the repair-associated markers (CD206 and CD163) and the pro-inflammatory-associated marker (CD86). (D) Densitometric quantification of CD206, CD163, and CD86 expression normalized to β-actin. (E) Immunofluorescence staining of repair-associated Arg-1 and pro-inflammatory-associated iNOS, IL-6, and TNF-α in peri-implant tissues from the indicated groups at 6 weeks after implantation. Arg-1 is shown in green, iNOS in red, IL-6 in magenta, TNF-α in yellow, and nuclei are stained with DAPI (blue). (F) Relative mRNA expression of *Arg1*, *Nos2*, *Il6*, and *Tnf* in peri-implant tissues from the indicated groups at 6 weeks after implantation. For quantitative analyses (B, D, F), *n* = 5 animals per group; data are presented as mean ± SD. Statistical comparisons among groups were performed using one-way ANOVA followed by Tukey's multiple-comparison test. Each protein or gene was analyzed separately. ns, not significant; *P < 0.05; **P < 0.01; ***P < 0.001; ****P < 0.0001. (For interpretation of the references to color in this figure legend, the reader is referred to the Web version of this article.)

The superior antibacterial performance of CUR-P-PUU compared with CUR/P-PUU may be related to the different modes of CUR incorporation and availability. In physically blended CUR/P-PUU, free CUR can diffuse out rapidly and may be partially lost from the defect region in the dynamic *in vivo* environment, leading to early depletion and reduced local antibacterial durability. In contrast, covalent integration retains CUR within the CUR-P-PUU polymer structure and, based on the *in vitro* degradation/availability results, provides more sustained degradation-associated CUR availability. The lower bacterial burden measured at 1 week and the less inflammatory, more repair-supportive immune profile observed at 6 weeks are consistent with this material design, although *in vivo* CUR availability was not directly quantified. Accordingly, the *in vivo* antibacterial data support an effect of CUR incorporation mode on local bacterial burden at the measured time point, rather than demonstrating sustained infection control or prevention of recolonization.

The immune microenvironment was further evaluated at 6 weeks after implantation. Western blot analysis showed reduced expression of CD86, a pro-inflammatory-associated macrophage marker, together with increased expression of CD206 and CD163, which are commonly associated with reparative macrophage phenotypes, in the CUR-P-PUU group (Fig. 8C and D). Immunofluorescence and RT–qPCR analyses showed consistent changes, including increased Arg-1 and *Arg1* expression, reduced iNOS and *Nos2* expression, and decreased IL-6 and TNF-α signals and transcripts (Fig. 8E and F; Supplementary Fig. 8A–D). Collectively, these findings indicate that CUR-P-PUU was associated with a less inflammatory and more repair-supportive local immune phenotype at 6 weeks after implantation.

These results should be interpreted within the dynamic immune environment of infected bone repair rather than as evidence of a simple binary M1-to-M2 transition. Residual bacterial burden, tissue injury, oxidative stress, scaffold degradation, and ongoing tissue remodeling can continuously influence macrophage responses over the course of healing. The marker profile measured here therefore represents the immune state at a defined postoperative time point rather than a temporally resolved polarization trajectory. Within this context, the lower inflammatory-marker expression and higher repair-associated marker expression observed with CUR-P-PUU are consistent with a local immune environment that is more supportive of tissue repair. However, longitudinal analyses at multiple implantation time points would be required to define the temporal evolution of macrophage phenotypes during regeneration.

Taken together, the *in vivo* antibacterial and immunomodulatory findings provide a biological context for the improved bone regeneration observed in CUR-P-PUU. Compared with physically blended CUR/P-PUU, CUR-P-PUU showed a more sustained, degradation-associated CUR-availability profile, which was accompanied by lower bacterial burden and a less inflammatory, more pro-reparative local immune phenotype. These findings support the central design principle of this study: covalent incorporation changes CUR from a separately loaded cargo into a functional component of the scaffold-forming polymer, thereby linking its mode of incorporation with material behavior and the duration of CUR-related bioactivity.

### 2.8 Limitations and translational considerations

The present study establishes CUR-P-PUU as a proof of concept, but several limitations remain. First, extension of this “bioactive-molecule-as-building-block” strategy beyond CUR will be chemistry-dependent and will require molecule-specific validation of reactivity, polymerization compatibility, biological stability, and effects on material properties and printability.

Second, although CUR-P-PUU was successfully processed into homogeneous inks and 3D-printed with preserved scaffold fidelity and interconnected porosity, manufacturing reproducibility and scalability were not systematically evaluated. Future studies should assess batch-to-batch polymer consistency, ink stability and rheology, printing reproducibility at larger scales, and the robustness of post-print processing and sterilization.

Third, the CUR content used here was selected mainly according to polymerization stoichiometry and scaffold processability rather than biological dose optimization. Because CUR functions as both a bioactive molecule and a reactive chain-extending component, changing the CUR/piperazine ratio may simultaneously affect polymer structure, printability, mechanics, degradation, and CUR availability. Future work should therefore identify a practical composition window that balances material performance with biological efficacy.

Finally, *in vivo* scaffold degradation and local CUR or CUR-derived species were not directly quantified, and the present biological assays do not define specific molecular mechanisms. The macrophage and cytokine data indicate changes in the local immune phenotype, the ABTS assay reflects radical-scavenging capacity, and the antibacterial and osteogenic results demonstrate functional outcomes rather than pathway-specific mechanisms. Further mechanistic and preclinical studies will therefore be required before clinical translation.

## 3 Conclusions

In this study, we developed a scaffold-as-drug strategy by covalently integrating CUR into a 3D-printable piperazine-based PUU backbone. By using CUR as a reactive phenolic chain extender and piperazine as a urea-forming structural chain extender, CUR was converted from a physically loaded cargo into a structural and functional component of the scaffold-forming polymer. This design preserved printability and interconnected porosity, improved apparent wettability and mechanical performance, and provided sustained, degradation-associated CUR availability. Although physically blended CUR/P-PUU showed stronger short-term osteogenic responses *in vitro*, CUR-P-PUU provided a more gradual CUR-availability profile and showed antibacterial, radical-scavenging, immunomodulatory, and osteogenic activities. In the rat MRSA-infected calvarial defect model, CUR-P-PUU reduced bacterial burden, was associated with a less inflammatory and more pro-reparative local immune phenotype, and enhanced bone formation and mineralization. Overall, these findings support covalent CUR integration as a promising materials-design strategy for combining structural and therapeutic functions within a biodegradable scaffold.

## 4 Experimental section

### 4.1 Synthesis of CUR-P-PUU polymers

CUR-P-PUU was synthesized through a two-step prepolymerization and chain-extension strategy. Briefly, PCL-diol (Mn = 2000; Sigma-Aldrich, St. Louis, MO, USA) was vacuum-dried before use to remove residual moisture. The dried PCL-diol was reacted with LDI (Tokyo Chemical Industry Co., Tokyo, Japan) under a dry nitrogen atmosphere at 80°C for 4 h to obtain an isocyanate-terminated polyurethane prepolymer. CUR (Shanghai Yuanye Bio-Technology Co., Shanghai, China) and piperazine (Shanghai Yuanye Bio-Technology Co., Shanghai, China) were then used as dual chain extenders to generate the CUR-integrated piperazine-based PUU polymer.

For CUR incorporation, CUR was first dissolved in anhydrous dimethyl sulfoxide (DMSO) under light-protected conditions and then added to the prepolymer solution. The reaction was allowed to proceed at 40°C for 12 h under a nitrogen atmosphere and protected from light to promote the reaction between the phenolic hydroxyl groups of CUR and the residual isocyanate (NCO) groups of the prepolymer. Subsequently, piperazine, separately dissolved in DMSO, was added dropwise to the reaction mixture to further consume the remaining NCO groups and complete the urea-forming chain-extension reaction. The final reactant concentration in the reaction mixture was adjusted to 4%. The reaction was continued at 40°C for an additional 12 h under nitrogen and light-protected conditions, giving a total chain-extension time of 24 h. Unless otherwise stated, the feed molar ratio of PCL-diol:LDI:piperazine:CUR was fixed at 1:2:0.75:0.25. Based on the nominal functionalities of piperazine and CUR, the active-hydrogen equivalents provided by the two chain extenders were designed to stoichiometrically match the residual NCO equivalents in the prepolymer.

After completion of the polymerization, the crude reaction mixture was first treated with methanol three times to remove unreacted or noncovalently associated CUR and other methanol-soluble small molecules. Deionized water was subsequently added to the reaction mixture to induce precipitation of the polymer. The precipitated polymer was collected and thoroughly washed with deionized water to remove residual DMSO, methanol, piperazine, and other water-soluble impurities. The purified CUR-P-PUU polymer was then vacuum-dried at 40°C to constant mass.

Pristine P-PUU was synthesized using the same prepolymerization procedure, except that CUR was omitted and piperazine was used as the sole chain extender. For this formulation, the molar ratio of PCL-diol:LDI:piperazine was adjusted to 1:2:1.00 to maintain stoichiometric balance between residual NCO groups and active hydrogen equivalents.

### 4.2 Chemical and molecular characterization of CUR-P-PUU polymers

#### 4.2.1 Molecular dynamics simulations

Molecular dynamics simulations were performed to compare the chain-level behavior and intermolecular interactions of pristine P-PUU and CUR-P-PUU. Polymer models were constructed according to the experimentally designed compositions, and all simulations were conducted using the COMPASS II force field. After initial geometry optimization, each system was equilibrated and subjected to dynamic simulation using the Forcite module. Trajectory analyses were performed to obtain mean squared displacement curves for assessing chain mobility and radial distribution function curves for evaluating interchain packing and local molecular ordering. Temperature and pressure were controlled using Andersen thermostat and barostat algorithms, and molecular motion was integrated using the velocity Verlet algorithm with a time step of 1 fs. Each system was equilibrated over the temperature range of 300–500 K for 500 ps, and the final equilibrated structures were used for subsequent interaction and trajectory analyses.

#### 4.2.2 Chemical characterization

Attenuated total reflectance ATR-FTIR spectroscopy was used to characterize the formation of urethane/urea linkages and to evaluate the incorporation of CUR into the P-PUU structure. LDI, PCL-diol, pristine P-PUU, and CUR-P-PUU samples were recorded using a Nicolet iS50 FTIR spectrometer (Thermo Fisher Scientific) equipped with an ATR accessory over the wavenumber range of 4000-600 cm^−1^, with a spectral resolution of 4 cm^−1^ and 32 scans per sample. The disappearance of the characteristic isocyanate absorption band of LDI, together with the appearance or enhancement of urethane/urea carbonyl and amide-related bands, was used to confirm completion of the chain-extension reaction. Changes in CUR-related aromatic and phenolic hydroxyl absorption bands were further analyzed to support CUR participation in urethane bond formation.

^1^H NMR spectroscopy was used to further confirm the molecular structure of CUR-P-PUU. CUR-P-PUU elastomer was dissolved in 0.6 mL of DMSO-d6 containing 0.03% tetramethylsilane. Spectra were acquired at 25°C using a Bruker AVANCE 400 MHz spectrometer (Bruker, Fällanden, Switzerland), with tetramethylsilane used as the internal reference. The successful incorporation of CUR was evaluated by the appearance of characteristic CUR-derived aromatic/olefinic proton signals, together with characteristic signals from PCL, LDI-derived segments, and piperazine. The attenuation or disappearance of free phenolic hydroxyl signals was used as supporting evidence for the reaction between CUR phenolic hydroxyl groups and residual NCO groups.

XPS was performed using a Thermo Scientific ESCALAB 250Xi system (Thermo Fisher Scientific, USA) equipped with a monochromatic Al Kα X-ray source (hν = 1486.6 eV). Survey spectra were collected with a pass energy of 100 eV, and high-resolution spectra of C 1s, O 1s, and N 1s were acquired with a pass energy of 50 eV. All binding energies were charge-corrected by referencing the adventitious carbon C 1s peak to 284.8 eV. The obtained spectra were used to evaluate the surface elemental composition and bonding environments of P-PUU and CUR-P-PUU.

GPC was performed to determine the molecular-weight distribution of P-PUU and CUR-P-PUU polymers. Polymer samples were dissolved in tetrahydrofuran (THF) at a concentration of 2 mg mL^−1^ and filtered through a 0.22 μm PTFE syringe filter before analysis. GPC measurements were carried out using a Waters 1515 gel permeation chromatograph equipped with a Waters 2414 refractive index detector and Styragel HR columns. THF was used as the mobile phase at a flow rate of 1.0 mL min^−1^, and the column temperature was maintained at 35°C. The system was calibrated using narrow-distribution polystyrene standards. Number-average molecular weight (Mn), weight-average molecular weight (Mw), and molecular-weight distribution were calculated from the chromatograms.

#### 4.2.3 Quantification of residual extractable CUR

To determine the amount of noncovalently retained CUR remaining in the purified CUR-P-PUU polymer, accurately weighed CUR-P-PUU samples (20 mg) were extracted with 2 mL of HPLC-grade methanol under light-protected conditions. The samples were sonicated for 30 min and subsequently centrifuged at 10,000 × g for 10 min. The supernatant was collected, and the extraction procedure was repeated three times to achieve exhaustive recovery of extractable CUR. The three supernatants were combined and adjusted to a final volume of 10 mL with methanol, filtered through a 0.22 μm PTFE syringe filter, and subjected to HPLC analysis.

CUR was quantified using an Agilent 1260 HPLC system equipped with a C18 reversed-phase column (4.6 × 250 mm, 5 μm). The mobile phase consisted of acetonitrile and water containing 0.1% formic acid at a ratio of 50:50 (v/v) under isocratic elution, at a flow rate of 1.0 mL min^−1^. The detection wavelength was set at 425 nm, and the column temperature was maintained at 30°C. The injection volume was 20 μL. CUR concentration was determined using an external calibration curve prepared in methanol over the range of 0.5–20 μg mL^−1^. Residual extractable CUR was expressed as the mass of extracted CUR relative to the dry mass of the purified polymer.

#### 4.2.4 Method validation

Calibration linearity was evaluated using CUR standards at 0.5, 1, 2, 5, 10, and 20 μg mL^−1^. The calibration equation and coefficient of determination (R^2^) were obtained by least-squares linear regression. Extraction recovery was evaluated by spiking blank P-PUU polymer with known amounts of CUR at low, medium, and high concentration levels, with each level analyzed in triplicate. Intra-assay precision was expressed as the relative standard deviation (RSD). The limits of detection and quantification were determined using signal-to-noise ratios of approximately 3 and 10, respectively.

### 4.3 Fabrication of 3D-printed porous scaffolds

P-PUU and CUR-P-PUU elastomers were dissolved in anhydrous DMSO at 50% (w/v) to prepare printable inks. For physically blended CUR/P-PUU, pristine P-PUU was dissolved in anhydrous DMSO at 50% (w/v), and CUR was added at a P-PUU:CUR mass ratio of 97:3 (w/w). All formulations were thoroughly mixed, sonicated to obtain homogeneous inks, and degassed before extrusion. No covalent reaction or post-blending polymer purification was performed for CUR/P-PUU.

Porous scaffolds were fabricated by layer-by-layer direct ink writing using a customized syringe-based extrusion module mounted on a desktop 3D printer (Ultimaker S5, Ultimaker, The Netherlands). The highly concentrated polymer ink (50% w/v) was loaded into a syringe barrel equipped with a 22-gauge (22G) flat-tip stainless-steel extrusion nozzle. The nozzle temperature was maintained at 80°C to reduce extrusion resistance and facilitate stable and continuous filament deposition. Owing to the high polymer concentration, the ink possessed sufficient consistency and polymer-chain entanglement to maintain short-term filament integrity after extrusion. Upon deposition onto a platform maintained at 40°C, cooling of the extruded filaments promoted shape retention and helped minimize filament spreading and collapse during successive layer deposition. The printing speed was set to 50 mm s^−1^, the layer thickness to 0.2 mm, and the infill density to 30%. An alternating 0°/90° laydown pattern was used to generate a regular and interconnected porous architecture.

Immediately after printing, the constructs were subjected to sequential ethanol/water solvent exchange to progressively remove DMSO and further stabilize the printed porous architecture. The scaffolds were immersed in fresh ethanol three times for 30 min each, followed by rinsing with deionized water three times for 30 min each under gentle agitation, and subsequently vacuum-dried at 40°C to constant mass. For CUR/P-PUU, CUR was physically incorporated into the P-PUU matrix before printing; therefore, partial loss of noncovalently retained CUR during solvent exchange could occur.

For *in vitro* experiments, dried scaffolds were sterilized by ultraviolet irradiation and rinsed with sterile PBS before use. Scaffolds intended for *in vivo* implantation were sterilized with ethylene oxide and sufficiently degassed before implantation, as described in Section 4.10. All scaffolds underwent the same solvent-exchange and vacuum-drying procedures before the respective sterilization treatment.

### 4.4 Characterization of 3D-printed scaffolds

#### 4.4.1 Gross morphology and scanning electron microscopy

Representative photographs of 3D-printed P-PUU and CUR-P-PUU scaffolds were captured using a Nikon digital single-lens reflex camera to evaluate macroscopic appearance and print fidelity. The surface and cross-sectional morphologies of the scaffolds were examined by SEM. Scaffold specimens were cut into approximately 1 mm^3^ blocks, mounted on conductive adhesive tapes, and sputter-coated with a thin gold layer of approximately 5 nm. SEM images were acquired using a Hitachi SU8010 field-emission SEM (Hitachi, Tokyo, Japan) at an accelerating voltage of 5 kV.

#### 4.4.2 Porosity, wettability, and water uptake

The porosity of 3D-printed P-PUU, physically blended CUR/P-PUU, and CUR-P-PUU scaffolds was determined using an ethanol displacement method. The dry mass of each scaffold was recorded as m_1_. The scaffolds were then immersed in ethanol under vacuum until no air bubbles were observed, indicating complete infiltration of ethanol into the interconnected pores. After gently removing surface ethanol with filter paper, the ethanol-saturated mass was recorded as m_2_. Scaffold porosity was calculated according to the following equation:

$$\text{Porosity}\;\left(\%\right)=\left(m_{2}-m_{1}\right)/\rho V\times 100\%$$

where ρ is the density of ethanol at 25°C, 0.789 g cm^−3^, and V is the apparent scaffold volume calculated from the geometric dimensions.

Static water contact angles were measured to evaluate the apparent surface wettability of the 3D-printed scaffolds. A water droplet of fixed volume was gently placed on the top printed surface of each scaffold, and images were captured within the same time window after droplet deposition. Contact angles were analyzed using contact-angle analysis software. Five independent scaffold specimens were analyzed per group, and all groups were tested under identical conditions to allow relative comparison among P-PUU, physically blended CUR/P-PUU, and CUR-P-PUU scaffolds.

Water uptake was evaluated by immersing pre-dried scaffolds in PBS at 37°C. The initial dry mass was recorded as W_0_. At predetermined time points, samples were removed from PBS, gently blotted with filter paper to remove surface water, and immediately weighed to obtain the wet mass, denoted as W_t_. Water uptake was calculated according to the following equation:

$$\text{Water}\;\text{uptake}\;\left(\%\right)=\left[\left(W_{t}-W_{0}\right)/W_{0}\right]\times 100\%$$

#### 4.4.3 TGA

The thermal stability of 3D-printed P-PUU and CUR-P-PUU scaffolds was evaluated by TGA using a Netzsch STA 449 F5 thermal analyzer (Netzsch, Selb, Germany). Briefly, approximately 5–10 mg of each dried scaffold sample was placed in an alumina crucible and heated from 30 to 800°C at a heating rate of 10°C min^−1^ under a nitrogen flow of 50 mL min^−1^. TGA curves were recorded to determine the thermal weight-loss behavior, and DTG curves were generated to compare the main thermal decomposition stages and maximum degradation temperatures of the scaffolds.

#### 4.4.4 Mechanical testing

The mechanical properties of 3D-printed P-PUU and CUR-P-PUU scaffolds were measured using an Instron 5967 universal testing machine (Instron, Norwood, MA, USA). For compression testing, cylindrical scaffolds with dimensions of Φ5 mm × 5 mm were compressed at a crosshead speed of 2 mm min^−1^ until 50% strain was reached. Cyclic compression testing was also performed to assess elastic recovery. The compressive modulus was calculated from the initial linear region of the stress–strain curve. For tensile testing, rectangular scaffold specimens (30 mm × 5 mm × 1 mm; length × width × thickness) were used, with an initial gauge length of 20 mm. The specimens were stretched at a crosshead speed of 5 mm min^−1^ until failure. Engineering stress was calculated based on the initial cross-sectional area, and tensile strength and elongation at break were obtained from the corresponding stress–strain curves. All mechanical tests were performed at 37°C after pre-equilibration of the scaffolds in PBS to better approximate physiological conditions.

#### 4.4.5 *In vitro* degradation and CUR release

The *in vitro* degradation behavior of 3D-printed P-PUU, physically blended CUR/P-PUU, and CUR-P-PUU scaffolds was evaluated under both hydrolytic and enzymatic conditions. Pre-dried cylindrical scaffolds with dimensions of Φ5 mm × 5 mm were weighed to record the initial dry mass, denoted as m_0_. The samples were then incubated at 37°C under shaking at 100 rpm in either PBS (pH 7.4) for hydrolytic degradation or 50 mM Tris-HCl buffer (pH 7.4) containing 0.1 mg mL^−1^ proteinase K (≥99%, Sigma-Aldrich) for enzymatic degradation. At each predetermined time point, the scaffolds were removed from the degradation medium, gently rinsed with deionized water to remove soluble degradation products and salts, and then vacuum-dried at 40°C until a constant mass was reached. The cumulative mass loss was calculated according to the following equation:

$$\text{Cumulative}\;\text{mass}\;\text{loss}\;\left(\%\right)=\left[\left(m_{0}-m_{t}\right)/m_{0}\right]\times 100\%$$

where m_0_ is the initial dry mass and m_t_ is the remaining dry mass after degradation for the indicated time period.

The release behavior of CUR from CUR-P-PUU and physically blended CUR/P-PUU scaffolds was quantified using HPLC. Each pre-dried cylindrical scaffold (Φ5 mm × 5 mm) was individually immersed in 5 mL of release medium in a light-protected container and incubated at 37°C under shaking at 100 rpm. For the enzymatic release experiment, the release medium consisted of 50 mM Tris-HCl buffer (pH 7.4) containing 0.1 mg mL^−1^ proteinase K and 0.2% (w/v) SDS. SDS was included to improve CUR solubilization and help maintain sink conditions. The entire release medium was collected every 3 days and immediately replaced with an equal volume (5 mL) of freshly prepared medium containing the same concentrations of proteinase K and SDS. All collected release media were protected from light, centrifuged to remove suspended scaffold debris, and filtered through a 0.22 μm filter before HPLC analysis. CUR concentration was determined using an Agilent 1260 HPLC system (Agilent Technologies, Santa Clara, CA, USA) equipped with a C18 reverse-phase column (4.6 × 250 mm, 5 μm). The mobile phase consisted of acetonitrile and water containing 0.1% formic acid at a flow rate of 1.0 mL min^−1^. The detection wavelength was set at 425 nm, and the column temperature was maintained at 30°C. CUR concentration was quantified using an external calibration curve prepared from standard CUR solutions. The cumulative amount of released CUR at each time point was calculated by summing the amount of CUR recovered in all previously collected release media, according to​$M_{t}=\sum_{i=1}^{n}C_{i}V$, where *C*_*i*_ is the CUR concentration in the collected medium at the ith sampling interval and *V* is the total medium volume (5 mL). Cumulative CUR release was expressed as a percentage of the initial CUR amount used for release normalization. Cumulative release values at 2, 4, 6, 8, 10, and 12 weeks were used for presentation and subsequent degradation–release association analysis.

For the enzymatic degradation/release experiment used for the degradation–release association analysis, scaffolds were incubated in 50 mM Tris-HCl buffer (pH 7.4) containing 0.1 mg mL^−1^ proteinase K and 0.2% (w/v) SDS. The same experimental condition and matched time points were used for cumulative mass-loss and CUR-release measurements. Cumulative CUR release (%) was plotted as a function of cumulative enzymatic mass loss (%), and linear regression analysis was performed. The coefficient of determination (R^2^) was used to describe the strength of the association between the two variables. This analysis was intended as a descriptive assessment of mass-loss-associated CUR availability and was not used to establish a specific molecular degradation or bond-cleavage mechanism.

### 4.5 *In vitro* cytocompatibility evaluation

Passage 3 Sprague–Dawley rat BMSCs (Shanghai Zhong Qiao Xin Zhou Biotechnology Co., Shanghai, China) were cultured in α-minimum essential medium (α-MEM; Gibco, Grand Island, NY, USA) supplemented with 10% fetal bovine serum, 100 U mL^−1^ penicillin, and 100 μg mL^−1^ streptomycin. Cells were maintained at 37°C in a humidified incubator containing 5% CO_2_, and the culture medium was refreshed every 2 days. Before cell seeding, 3D-printed P-PUU and CUR-P-PUU scaffolds were sterilized and pre-equilibrated in complete culture medium. BMSCs were seeded onto the scaffolds, and BMSCs cultured without scaffolds under the same conditions served as the control.

Cell viability was evaluated after 1, 3, 5, and 7 days of culture using a Live/Dead staining kit (Invitrogen, USA). Fluorescence images were captured using an Olympus BX53 fluorescence microscope (Olympus, Tokyo, Japan). The numbers of live and dead cells were quantified using ImageJ software, and cell viability was calculated as the percentage of live cells relative to the total number of cells.

BMSC proliferation was assessed using a CCK-8 assay (Dojindo, Kumamoto, Japan). BMSCs were seeded onto sterilized scaffolds placed in 96-well plates at a density of 5 × 10^3^ cells per well. Cells seeded directly onto tissue-culture polystyrene at the same density served as the control. At 1, 3, 5, and 7 days, 10 μL of CCK-8 reagent was added to each well and incubated for 2 h at 37°C. The absorbance of the supernatant was measured at 450 nm using a Tecan Spark microplate reader (Tecan, Männedorf, Switzerland).

Cell proliferation was further evaluated by quantifying total DNA content. At 1, 3, 5, and 7 days, cells associated with the scaffolds were collected and lysed, while control cells were processed in parallel. DNA content was measured using the PicoGreen dsDNA assay kit (Thermo Fisher Scientific) according to the manufacturer's protocol. Fluorescence intensity was recorded at an excitation wavelength of 480 nm and an emission wavelength of 520 nm, and DNA concentration was calculated using a standard curve.

Cell adhesion and spreading were assessed by cytoskeletal staining. After 1, 3, 5, and 7 days of culture, cells in each group were fixed with 4% paraformaldehyde, permeabilized with 0.1% Triton X-100, and stained with TRITC-phalloidin (1:200; Sigma-Aldrich) to visualize F-actin. Cell nuclei were counterstained with DAPI (1 μg mL^−1^; Sigma-Aldrich). Fluorescence images were acquired using a Zeiss Axio Observer 7 microscope (Zeiss, Jena, Germany).

### 4.6 *In vitro* anti-inflammatory evaluation

The anti-inflammatory activity of CUR-containing scaffolds was evaluated using scaffold extracts in LPS-stimulated RAW264.7 macrophages. RAW264.7 cells (ATCC TIB-71) were cultured in high-glucose Dulbecco's modified Eagle medium (DMEM; Gibco, Grand Island, NY, USA) supplemented with 10% fetal bovine serum at 37°C in a humidified atmosphere containing 5% CO_2_. Prior to the assay, extracts of P-PUU, physically blended CUR/P-PUU, and CUR-P-PUU scaffolds were prepared by incubating sterilized scaffolds in serum-free high-glucose DMEM at 37°C for 24 h using an extraction ratio of 0.1 g mL^−1^. The collected extracts were centrifuged to remove debris, filtered through 0.22 μm sterile filters, supplemented with 10% fetal bovine serum, and used immediately for cell treatment.

For inflammatory stimulation, RAW264.7 macrophages were seeded in culture plates and allowed to adhere overnight. The cells were then stimulated with LPS (1 μg mL^−1^; Sigma-Aldrich) for 6 h to establish an inflammatory macrophage model. After LPS stimulation, the medium was replaced with the corresponding treatment medium. The experimental groups were as follows: Control, macrophages cultured in complete medium without LPS stimulation; LPS, macrophages stimulated with LPS and then cultured in complete medium; CUR + LPS, LPS-stimulated macrophages treated with free CUR at 5 μM; P-PUU + LPS, LPS-stimulated macrophages treated with P-PUU scaffold extract; CUR/P-PUU + LPS, LPS-stimulated macrophages treated with physically blended CUR/P-PUU scaffold extract; and CUR-P-PUU + LPS, LPS-stimulated macrophages treated with CUR-P-PUU scaffold extract. All treatments were continued for 24 h. For the free CUR group, CUR was dissolved in DMSO before dilution in culture medium, and the final DMSO concentration was maintained at 0.1% v/v in all relevant groups.

For immunofluorescence staining, macrophages were fixed with 4% paraformaldehyde, permeabilized with 0.1% Triton X-100, and blocked with bovine serum albumin-containing blocking buffer. The cells were then incubated overnight at 4°C with primary antibodies against IL-6, IL-1β, or TNF-α (each 1:200; Abcam). After washing with PBS, the samples were incubated with fluorophore-conjugated secondary antibodies (1:500; Invitrogen), and nuclei were counterstained with 4′,6-diamidino-2-phenylindole (DAPI). Fluorescence images were acquired using a Zeiss LSM 980 confocal microscope, and the mean fluorescence intensity of each inflammatory marker was quantified using ImageJ software.

Macrophage morphology was further assessed by cytoskeletal staining. Briefly, F-actin was stained with TRITC-phalloidin, and nuclei were counterstained with DAPI to evaluate LPS-induced morphological activation and the regulatory effects of CUR-containing scaffold extracts on macrophage spreading and cytoskeletal organization.

### 4.7 *In vitro* antibacterial evaluation

The antibacterial activity of the scaffolds was evaluated against MRSA (ATCC 43300). Sterilized 3D-printed scaffolds with defined dimensions of 5 mm in diameter and 2 mm in thickness were placed in 24-well plates and incubated with 1 mL of MRSA suspension at an initial concentration of 1 × 10^6^ CFU mL^−1^. The experimental groups included bacteria-only control, free CUR, P-PUU, physically blended CUR/P-PUU, and CUR-P-PUU. For the free CUR group, CUR was first dissolved in DMSO and then diluted in bacterial culture medium to a final concentration of 5 μM. The final DMSO concentration was maintained at 0.1% v/v in all DMSO-containing groups.

After incubation at 37°C for 12 and 24 h, the planktonic bacterial suspension from each well was collected. Aliquots were used for CFU counting and live/dead bacterial staining. For CFU analysis, the planktonic suspensions were serially diluted, plated onto LB agar plates, and incubated at 37°C for 24 h. Viable planktonic bacteria were quantified by colony counting.

Live/dead bacterial staining was performed to evaluate the viability of planktonic MRSA after co-culture with the different scaffold formulations. Aliquots of the planktonic bacterial suspensions collected from each group at 12 and 24 h were stained using a Live/Dead BacLight bacterial viability kit containing SYTO 9 and propidium iodide according to the manufacturer's instructions. Fluorescence images were captured using an Olympus BX53 fluorescence microscope, and the live/dead bacterial ratio was quantified using ImageJ software.

### 4.8 *In vitro* radical-scavenging activity assay

The radical-scavenging activity of degradation media collected from P-PUU, physically blended CUR/P-PUU, and CUR-P-PUU scaffolds was evaluated using an ABTS radical cation-scavenging assay. ABTS stock solution (7 mM) was mixed with potassium persulfate solution (2.45 mM) at a volume ratio of 1:1 and incubated in the dark at room temperature for 12 h to generate ABTS•^+^ radicals. Before use, the ABTS•^+^ solution was diluted with ethanol to obtain a working solution with an absorbance of 0.70 ± 0.02 at 734 nm.

For the assay, 100 μL of degradation supernatant from each scaffold group was mixed with 1.9 mL of ABTS•^+^ working solution and incubated for 15 min in the dark at room temperature. The absorbance was then measured at 734 nm using a Shimadzu UV-2600 spectrophotometer (Shimadzu, Kyoto, Japan). The corresponding degradation medium without scaffold treatment was used as the control to determine the initial absorbance, denoted as A_0_. ABTS radical-scavenging efficiency was calculated according to the following equation:

$$\text{Scavenging}\;\text{efficiency}\;\left(\%\right)=\left[\left(A_{0}-A_{s}\right)/A0\right]\times 100\%$$

where A_s_ is the absorbance of the sample after reaction with the ABTS•^+^ working solution.

### 4.9 *In vitro* osteogenic differentiation evaluation

BMSCs were used to evaluate the pro-osteogenic effects of the scaffolds. BMSCs were cultured with the indicated scaffolds in osteogenic induction medium. The experimental groups were Control, P-PUU, physically blended CUR/P-PUU, and CUR-P-PUU.

For ALP staining, BMSCs were cultured under osteogenic induction for 7 days. The cells were fixed and stained using an ALP staining kit according to the manufacturer's instructions. ALP-positive staining was imaged, and ALP activity was quantitatively measured and normalized to total protein content.

For matrix mineralization analysis, BMSCs were cultured under osteogenic induction for 21 days. The cells were fixed and stained with ARS to detect calcium deposition and mineralized matrix formation. Matrix-bound ARS was then extracted with 10% (w/v) cetylpyridinium chloride (CPC) in 10 mM sodium phosphate buffer (pH 7.0) for 30 min at room temperature under gentle shaking. The extracts were transferred to 96-well plates, and absorbance was measured at 562 nm. ARS content was calculated using a standard curve and normalized to total protein content.

For western blot analysis, total proteins were extracted from cells in each group, separated by SDS–PAGE, and transferred onto PVDF membranes. After blocking, the membranes were incubated overnight at 4°C with primary antibodies against RUNX2 (1:1000), OCN (1:800), OPN (1:1000), COL I (1:1000) (all from Abcam), and β-actin (1:5000; Sigma-Aldrich). After incubation with horseradish peroxidase-conjugated secondary antibodies (1:5000; Invitrogen), protein bands were visualized using enhanced chemiluminescence reagent and quantified using ImageJ software. β-Actin was used as the internal loading control.

For RT–qPCR, total RNA was extracted from cells using TRIzol reagent (Invitrogen). RNA concentration and purity were determined using a NanoDrop 2000 spectrophotometer. The extracted RNA was reverse-transcribed into complementary DNA using a reverse transcription kit, followed by amplification using SYBR Green Mix on a QuantStudio 5 real-time PCR system (Thermo Fisher Scientific). β-Actin was used as the housekeeping gene, and the relative mRNA expression levels of *Runx2*, *Bglap*, *Spp1*, and *Col1a1* were calculated using the 2^−ΔΔCt^ method. Primer sequences are listed in Supplementary Table S2.

### 4.10 *In vivo* MRSA-infected bone defect repair evaluation

All animal procedures were approved by the Institutional Animal Care and Use Committee of Shanghai Ruijin Hospital, Shanghai Jiao Tong University School of Medicine, and were performed in accordance with institutional animal welfare guidelines and the principles of the 3Rs. Healthy male Sprague–Dawley rats, 8 weeks old and weighing 250–300 g, were used for the *in vivo* infected bone defect repair study.

Rats were anesthetized with inhaled isoflurane delivered in oxygen using an anesthesia vaporizer. Anesthesia was induced with 5% isoflurane and maintained at 2.5% during surgery. Adequate anesthetic depth was supported by the absence of the pedal withdrawal reflex, and body temperature was maintained using a thermostatically controlled heating pad throughout the procedure. After aseptic preparation, a midline incision was made over the calvarium, and the periosteum was carefully reflected to expose the parietal bones. Full-thickness calvarial defects (5 mm in diameter) were created in the parietal bones using a sterile trephine bur under continuous saline irrigation. To establish an infected bone defect model, 100 μL of MRSA suspension at a concentration of 1 × 10^6^ CFU mL^−1^ was introduced into each defect.

Animals were randomly assigned to four groups: Control, infected defects without scaffold implantation; P-PUU, infected defects implanted with pristine P-PUU scaffolds; CUR/P-PUU, infected defects implanted with physically blended CUR/P-PUU scaffolds; and CUR-P-PUU, infected defects implanted with covalently integrated CUR-P-PUU scaffolds. For implantation, cylindrical scaffolds with dimensions of Φ5 mm × 1 mm were fabricated, sterilized with ethylene oxide, sufficiently degassed, and placed into the defect sites. The periosteum and skin were then closed in layers. Animals were monitored postoperatively according to the approved animal care protocol.

At 6 and 12 weeks after implantation, rats were euthanized by overdose anesthesia, and the calvarial specimens were harvested and fixed in 4% paraformaldehyde for 24 h. Bone regeneration within the defect region was evaluated by micro-CT using a Bruker SkyScan 1276 system (Bruker, Kontich, Belgium) at 80 kV and 50 μA with a spatial resolution of 10 μm. The entire defect region was scanned, and DICOM datasets were reconstructed for 3D visualization. Quantitative analysis was performed using CTAn software, with the original 5 mm defect region defined as the region of interest. BV/TV and BMD were calculated to assess new bone formation and mineralization.

After micro-CT analysis, the specimens were decalcified in 10% ethylenediaminetetraacetic acid (EDTA, pH 7.4), dehydrated through graded ethanol, cleared, and embedded in paraffin. Serial sections with a thickness of 5 μm were prepared across the central region of the defects. H&E staining and Masson's trichrome staining were performed according to standard protocols to evaluate inflammatory cell infiltration, tissue organization, collagen deposition, and new bone formation. Histological images were acquired using a Zeiss Axio Observer 7 microscope.

### 4.11 *In vivo* antibacterial evaluation

The *in vivo* antibacterial efficacy of the scaffolds was evaluated at 1 week after implantation. Rats from each group were euthanized by overdose anesthesia, and the calvarial defect regions were surgically exposed under aseptic conditions. Peri-defect tissues from the Control group and peri-implant tissues from the P-PUU, CUR/P-PUU, and CUR-P-PUU groups were carefully harvested using sterile instruments while avoiding contamination from the surrounding skin, hair, or external environment. The collected tissues were homogenized in sterile PBS under aseptic conditions. The resulting tissue homogenates were serially diluted, plated onto LB agar plates, and incubated at 37°C for 24 h. Bacterial colonies were counted, and the local bacterial burden was expressed as CFU mL^−1^ of tissue homogenate and compared among groups to evaluate the *in vivo* antibacterial performance of the scaffolds.

### 4.12 *In vivo* immunomodulatory evaluation

At 6 weeks after implantation, *in vivo* inflammatory responses and macrophage phenotype-associated marker expression within the infected defect microenvironment were evaluated. Rats from each group were euthanized by overdose anesthesia, and the calvarial defect regions were surgically exposed under aseptic conditions. Peri-defect tissues from the Control group and peri-implant tissues from the P-PUU, CUR/P-PUU, and CUR-P-PUU groups were carefully harvested using sterile instruments, while avoiding contamination from the surrounding skin and hair. The collected tissues were immediately divided into aliquots for western blotting, immunofluorescence staining, and RT–qPCR analysis. Samples for protein and RNA extraction were processed on ice to minimize protein and RNA degradation.

For western blotting, total proteins were extracted from peri-defect or peri-implant tissues using protein lysis buffer containing protease inhibitors. Protein concentrations were determined using a standard protein assay, and equal amounts of protein from each sample were separated by SDS–PAGE on 10% gels. The proteins were then electrophoretically transferred onto PVDF membranes. After blocking, the membranes were incubated overnight at 4°C with primary antibodies against CD86 (a pro-inflammatory-associated macrophage marker; 1:1000; Sigma-Aldrich, HPA005417), CD206 (a repair-associated macrophage marker; 1:1000; Abcam, ab64693), CD163 (a repair-associated macrophage marker; 1:800; Abcam, ab182422), and β-actin ([actual dilution and source]) as the loading control. After washing, the membranes were incubated with appropriate horseradish peroxidase-conjugated secondary antibodies. Protein bands were visualized using an enhanced chemiluminescence detection system and quantified using ImageJ software. The expression levels of target proteins were normalized to β-actin.

For immunofluorescence staining, paraffin-embedded peri-implant tissue sections were deparaffinized, rehydrated, and subjected to antigen retrieval. After blocking with 5% bovine serum albumin, the sections were incubated overnight at 4°C with primary antibodies against Arg-1, iNOS, IL-6, or TNF-α. The sections were then incubated with the corresponding fluorophore-conjugated secondary antibodies. Different fluorescence channels were used for different markers: Arg-1 was visualized in green using Alexa Fluor 488, iNOS was visualized in red using Alexa Fluor 594, IL-6 was visualized in magenta using Alexa Fluor 647, and TNF-α was visualized in yellow/orange using Alexa Fluor 555. Nuclei were counterstained with DAPI. Fluorescence images were captured using a fluorescence microscope under identical exposure settings within each staining set, and mean fluorescence intensity was quantified using ImageJ software.

For RT–qPCR, total RNA was extracted from peri-defect or peri-implant tissues using TRIzol reagent according to the manufacturer's instructions. RNA concentration and purity were determined, and the extracted RNA was reverse-transcribed into complementary DNA. RT–qPCR was then performed using SYBR Green Mix to quantify the expression of macrophage phenotype-associated markers and inflammatory cytokines, including *Arg1*, *Nos2*, *Il6*, and *Tnf*. β-Actin was used as the housekeeping gene, and relative gene expression was calculated using the 2^−ΔΔCt^ method. Primer sequences are listed in Supplementary Table S2.

### 4.13 Statistical analysis

All quantitative data are presented as mean ± standard deviation (SD). Unless otherwise stated, quantitative experiments were performed with five independent experimental units per group (*n* = 5), consisting of independent scaffold specimens for material tests, independent biological replicates for *in vitro* biological assays, or individual animals for *in vivo* experiments, as appropriate. Comparisons between two groups were performed using an unpaired two-tailed Student's t-test. Comparisons among multiple groups at a single endpoint were performed using one-way ANOVA followed by Tukey's multiple-comparison test. Datasets involving treatment group and time were analyzed using two-way ANOVA followed by Šídák's multiple-comparison test. Linear regression was used for the degradation–CUR availability association analysis. Statistical significance was defined as follows: ns, not significant; *P < 0.05; **P < 0.01; ***P < 0.001; and ****P < 0.0001.

## CRediT authorship contribution statement

**Tao Yu:** Data curation, Conceptualization. **Jianxiang Zhu:** Funding acquisition, Formal analysis. **Chenguang Cai:** Methodology, Investigation. **Jiawei Mei:** Resources, Project administration. **Yong Xu:** Resources, Methodology. **Liying Yang:** Supervision, Software. **Xiangyang Xu:** Validation, Supervision. **Ming Ni:** Visualization, Validation. **Qianming Li:** Writing – review & editing, Writing – original draft.

## Declaration of competing interest

The authors declare that they have no known competing financial interests or personal relationships that could have appeared to influence the work reported in this paper.

## Data Availability

Data will be made available on request.

## Appendix A Supplementary data

Mutlimedia component
