# Supplementary material for: Covalently integrated curcumin turns piperazine-based polyurethane-urea into a scaffold-as-drug for infected bone repair
Source: Mater Today Bio. 2026 Oct 20;41:103763. doi: 10.1016/j.mtbio.2026.103763 (PMC13745765; doi:10.1016/j.mtbio.2026.103763)
Supplement: Supplementary file 1 [file mmc1.docx]

**Supporting Information**

**Covalently integrated curcumin turns piperazine-based polyurethane-urea into a scaffold-as-drug for infected bone repair**

*Tao Yu^1,2†^, Jianxiang Zhu^3†^, Chenguang Cai^4,5†^, Jiawei Mei^5†^, Yong Xu^7^, Liying Yang^6*^, Xiangyang Xu^1*^, Ming Ni^1*^, Qianming Li^5*^*

^1^ Department of Orthopaedics, Ruijin Hospital, Shanghai Jiao Tong University, School of Medicine, Shanghai, China;

^2^ Department of Orthopaedics, Ruijin-Hainan Hospital (Hainan Boao Research Hospital), Shanghai Jiao Tong University, School of Medicine, Qionghai, China;

^3^ Department of Orthopaedics, First Affiliated Hospital of Huzhou University, Huzhou, China;

^4^ Graduate School of Bengbu Medical University, Bengbu, China;

^5^ Department of Orthopedics, Centre for Leading Medicine and Advanced Technologies of IHM, The First Affiliated Hospital of USTC, Division of Life Sciences and Medicine, University of Science and Technology of China, Hefei, China;

^6^ Department of Nursing, Ruijin Hospital, Shanghai Jiao Tong University, School of Medicine, Shanghai, China;

^7^ Department of Thoracic Surgery, Shanghai Pulmonary Hospital, School of Medicine, Tongji University, Shanghai, China.

^†^ These authors contributed equally to the work.

* Corresponding authors: Prof. Liying Yang, Email: yly21248@rjh.com.cn; Dr. Xiangyang Xu, Email: xu664531@163.com; Dr. Ming Ni, Email: gendianqing@163.com; Dr. Qianming Li, Email: sweetleedemon@sina.com.

**Supplementary Figure 1.** Schematic illustration of the synthesis of CUR-P-PUU through covalent incorporation of CUR into the P-PUU backbone.

**Supplementary Figure 2.** High-resolution C 1s XPS spectra and deconvoluted peak components of P-PUU and CUR-P-PUU. The C 1s spectra were fitted into contributions corresponding to C–C/C–H, C–O, C=O, and N–COO species. CUR-P-PUU additionally exhibited a π–π* shake-up feature associated with aromatic/conjugated structures. The black curves represent the experimental spectra, the red curves represent the overall fitted envelopes, and the colored curves represent the individual fitted components.

**Supplementary Figure 3.** (A) Porosity, (B) static water contact angle, and (C) water uptake ratio of pristine P-PUU, physically blended CUR/P-PUU, and CUR-P-PUU scaffolds. *n* = 5 independent scaffold specimens per group; data are presented as mean ± SD. Statistical comparisons among groups were performed using one-way ANOVA followed by Tukey’s multiple-comparison test. ns, not significant; **P* < 0.05; ***P* < 0.01; ****P* < 0.001.

**Supplementary Figure 4. Cytoskeletal organization of RAW264.7 macrophages under inflammatory stimulation.** Representative fluorescence images of F-actin staining in RAW264.7 macrophages from the Control group without LPS stimulation and the LPS, P-PUU + LPS, CUR/P-PUU + LPS, and CUR-P-PUU + LPS groups. Nuclei were counterstained with DAPI.

**Supplementary Figure 5. ABTS radical-scavenging activity of degradation media from CUR-containing scaffolds.** (A) ABTS radical-scavenging efficiency of degradation media collected from pristine P-PUU, physically blended CUR/P-PUU, and CUR-P-PUU scaffolds. (B) Time-dependent ABTS radical-scavenging activity of degradation media from pristine P-PUU, physically blended CUR/P-PUU, and CUR-P-PUU scaffolds. *n* = 5 independent scaffold degradation samples per group; data are presented as mean ± SD. For panel A, statistical comparisons among groups were performed using one-way ANOVA followed by Tukey’s multiple-comparison test. For panel B, statistical comparisons were performed using two-way ANOVA followed by Šídák’s multiple-comparison test. ns, not significant; **P* < 0.05; *****P* < 0.0001.

**Supplementary Figure 6.** Schematic illustration of the establishment of an MRSA-infected bone defect rat model and implantation of the 3D-printed CUR-P-PUU scaffold into the defect region.

**Supplementary Figure 7. Micro-CT quantification of bone regeneration.** Quantitative analysis of (A) bone volume fraction (BV/TV) and (B) bone mineral density (BMD) in the control, P-PUU, CUR/P-PUU, and CUR-P-PUU groups at 6 and 12 weeks after implantation, based on micro-CT analysis. *n* = 5 animals per group at each time point; data are presented as mean ± SD. Statistical comparisons were performed using two-way ANOVA with treatment group and implantation time as factors, followed by Šídák’s multiple-comparison test. **P* < 0.05; ***P* < 0.01.

**Supplementary Figure 8. Semi-quantitative immunofluorescence analysis of *in vivo* immunomodulatory responses.** Semi-quantitative analysis of fluorescence intensity for (A) Arg-1, a repair-associated macrophage marker; (B) iNOS, a pro-inflammatory-associated macrophage marker; (C) IL-6, a pro-inflammatory cytokine; and (D) TNF-α, a pro-inflammatory cytokine, in peri-implant tissues from the control, P-PUU, CUR/P-PUU, and CUR-P-PUU groups. *n* = 5 animals per group; data are presented as mean ± SD. For each marker, statistical comparisons among groups were performed separately using one-way ANOVA followed by Tukey’s multiple-comparison test. ns, not significant; **P* < 0.05; *****P* < 0.0001.

**Supplementary Table S1.** Peak assignments of ^1^H NMR spectra for CUR-P-PUU.

| **Chemical shift** | **Assignment** | **Component** |
| --- | --- | --- |
| ~7.4, 7.1, 6.7 ppm | aromatic/olefinic protons | CUR |
| ~3.9, 2.3, 1.5, 1.3 ppm | methylene protons | PCL/LDI segment |
| ~3.8 ppm | Ester methoxy group | CUR/LDI segment |
| ~3.0 ppm | LDI-derived methylene protons | LDI segment |
| ~2.9 ppm | piperazine protons | Piperazine segment |

**Supplementary Table S2.** Primer sequences used for RT-qPCR.

| Genes | Primer direction | Primer sequence |
| --- | --- | --- |
| *Runx2* | Forward | 5’- ATGACACTGCCACCTCTGACTTCT -3’ |
|  | Reverse | 5’- AGGGATGAAATGCTTGGGAACT -3’ |
| *Bglap* | Forward | 5’- GAACAGACAAGTCCCACACAGC -3’ |
|  | Reverse | 5’- TCAGCAGAGTGAGCAGAAAGAT -3’ |
| *Spp1* | Forward | 5’- TGGCTGAATTCTGAGGGACTAA -3’ |
|  | Reverse | 5’- GCAGGCTGTAAAGCTTCTTCTCC -3’ |
| *Col1a1* | Forward | 5’- GAGAGGTGAACAAGGTCCCG -3’ |
|  | Reverse | 5’- AAACCTCTCTCGCCTCTTGC -3’ |
| *Arg1* | Forward | 5’- ACATTGGCTTGCGAGACGTA -3’ |
|  | Reverse | 5’- ATCACCTTGCCAATCCCCAG -3’ |
| *Nos2* | Forward | 5’- ACTCAGCCAAGCCCTCA -3’ |
|  | Reverse | 5’- CTCTGCCTATCCGTCTCGT -3’ |
| *Il6* | Forward | 5’- TAGTCCTTCCTACCCCAATTTCC -3’ |
|  | Reverse | 5’- TTGGTCCTTAGCCACTCCTTC -3’ |
| *Tnf* | Forward | 5’- CCCACGTCGTAGCAAACCAC -3’ |
|  | Reverse | 5’- ATGAGAGGGAGGCCATTTGG -3’ |
| *β-actin* | Forward | 5’- ACTTTGGGGGATGTTTGCTC -3’ |
|  | Reverse | 5’- GACTGTTACTGAGCTGCGTTT -3’ |
